# A multi-epitope vaccine targeting *Mycoplasma pneumoniae* P1 adhesin and CARDS toxin confers synergistic protection by adhesion inhibition and toxin neutralization

**DOI:** 10.3389/fimmu.2026.1930950

**Published:** 2026-09-22

**Authors:** Pengxiao Feng, Rui Hu, Huanbing Long, Jiarong He, Yuyao Xia, Jizhe Yang, Aihua Lei, Cuiming Zhu

**Affiliations:** Institute of Pathogenic Biology, Hengyang Medical College, University of South China, Hunan Provincial Key Laboratory for Special Pathogens Prevention and Control, Hengyang, Hunan, China

**Keywords:** community-acquired respiratory distress syndrome toxin, immune response, multi-epitope vaccine, *Mycoplasma pneumoniae*, P1 adhesin

## Abstract

**Background:**

*Mycoplasma pneumoniae* (Mp) remains a major cause of community-acquired pneumonia in children and adolescents, and no vaccine against this pathogen is currently available. This study designed and evaluated P1Tx, a novel multi-epitope vaccine candidate derived from the immunodominant regions of the P1 adhesin and the community-acquired respiratory distress syndrome (CARDS) toxin.

**Methods:**

Immunoinformatics approaches were used to select immunodominant epitopes from the P1 adhesin and the CARDS toxin of *Mp*, which were assembled into a 263-amino acid chimeric construct designated P1Tx. The construct was expressed in *Escherichia coli*, and the recombinant protein was characterized for physicochemical properties and predicted structural stability. Its immunoreactivity was assessed using sera from *Mp*-infected patients. BALB/c mice received three subcutaneous immunizations with P1Tx, and humoral and cellular immune responses were evaluated by antibody endpoint titers and flow cytometric analysis of germinal center B cells and T cell subsets. The functional activity of immune sera was tested by measuring its capacity to block *Mp* adhesion to human bronchial epithelial cells and to neutralize CARDS toxin-induced cytotoxicity. Protective efficacy was assessed in mice challenged intranasally with the *Mp* M129 strain, with pulmonary bacterial burden and histopathological changes determined after challenge.

**Results:**

P1Tx displayed favorable physicochemical characteristics and structural stability and reacted strongly with sera from *Mp*-infected patients. Immunized mice developed high-titer, IgG1-dominant antibody responses (endpoint titers up to 1:50,000) accompanied by marked germinal center B cell expansion. Compared with the P1C subunit vaccine, P1Tx elicited substantially stronger cellular immunity, with significant expansion of CD4+ and CD8+ effector and memory T cell populations. Sera from P1Tx-immunized mice inhibited *Mp* adhesion to bronchial epithelial cells and neutralized CARDS toxin-mediated cytotoxicity in vitro. Following intranasal challenge, P1Tx-immunized mice showed a 98.5% reduction in pulmonary bacterial burden and a 52.4% reduction in lung histopathological scores relative to the PBS group.

**Conclusions:**

P1Tx confers protection against *Mp* infection through complementary mechanisms of adhesion blockade and toxin neutralization, supporting its further development as a vaccine candidate for the prevention of *Mp* infection.

## Introduction

*Mycoplasma pneumoniae* (*Mp*), a cell wall-deficient microorganism with one of the smallest genomes (816 kb) among self-replicating pathogens, causes pneumonia, bronchopneumonia, and asthma via respiratory droplet transmission (1–4). Primarily affecting children and adolescents aged 3–18 years and immunocompromised individuals (5), it accounts for 10%-30% of pediatric pneumonia cases worldwide, rising to 30%-50% during epidemics (6–9). Clinical manifestations range from asymptomatic infection (30%-50%) to severe pneumonia with extrapulmonary complications (5). Antimicrobial resistance complicates the management of *Mp* pneumonia (MPP). Intrinsic resistance to β-lactams and widespread macrolide resistance in various regions limit therapeutic options (10, 11). Alternative agents like tetracyclines and fluoroquinolones carry risks of dental developmental toxicity in children and potential neuromuscular or cartilage impairment (12, 13), respectively. These challenges underscore the urgent need for *Mp* prophylactic vaccines.

Currently, there are no commercially available vaccines for *Mp*. Existing vaccine development platforms mainly include inactivated, live-attenuated, subunit, and nucleic acid-based approaches (14–16). Among these, subunit and nucleic acid vaccines primarily target microbial adhesins or the community-acquired respiratory distress syndrome (CARDS) toxin as key immunogenic components (14, 17–19). The adhesins of *Mp* include P1, P30, P116, P40/P90, and other accessory proteins. Notably, the P1 protein is the predominant and highly abundant *Mp* adhesin; specific antibodies against it significantly inhibit *Mp* adhesion and gliding motility (20, 21). However, the *p*1 gene contains two repeat regions, RepMP4 and RepMP2/3, which predispose it to recombination-induced variation (22). The adhesive and immunogenic regions of the P1 protein are mainly located within its C-terminus. Consequently, vaccine studies using the P1 adhesin as an antigen primarily target the amino acid sequence spanning residues 1107 to 1518 (23–25). Another key pathogenic factor, the CARDS toxin, characterized by its ADP-ribosyltransferase activity and vacuolating cytotoxicity, is a pivotal virulence factor that causes severe pulmonary inflammation in *Mp* pathogenesis (14, 17, 18). Its production correlates positively with the severity of pulmonary disease (26, 27). Mutations substituting glutamic acid with alanine at positions 269 and 271 (E269A/E271A) disrupt CARDS toxin intracellular retrograde transport to the endoplasmic reticulum, thereby abolishing its ADP-ribosyltransferase enzymatic activity and vacuolating cytotoxicity. These mutant toxins retain immunogenicity without cytotoxicity and have demonstrated preclinical efficacy in mice (28, 29).

While both the P1 adhesin and the CARDS toxin represent promising vaccine targets, no subunit vaccine co-targeting these two antigens has been reported to date, likely due to their distinct antigenic architectures that prevent soluble, stable co-folding in a single expression system, and to the empirical burden of balancing molar ratio, adjuvant, and schedule when the proteins are produced separately. The multi-epitope vaccine (MEV) platform offers a rational strategy to overcome these limitations by precisely integrating key B cell and T cell epitopes while excluding non-essential regions (30–33). In this study, we aimed to design and evaluate a novel MEV, designated P1Tx, which incorporates selected immunodominant epitopes from both the P1 adhesin and the CARDS toxin of *Mp*. Using a recombinant protein approach, we produced P1Tx and assessed its immunogenicity and protective efficacy in a murine model of *Mp* infection. Our findings demonstrate that vaccination with P1Tx elicits robust humoral and cellular immune responses, reduces bacterial load in the lungs, and attenuates infection-induced pulmonary inflammation and tissue injury. These results support the further development of P1Tx as a promising vaccine candidate against MPP.

## Materials and methods

### Study period

Human serum samples from MPP patients were collected between October 2023 and August 2024 from the Second Affiliated Hospital of the University of South China, Affiliated Nanhua Hospital of the University of South China, and Hengyang Central Hospital. The period for data collection and analysis of the human samples was from September 2024 to December 2024. The *in vivo* animal experiments were conducted between October 2024 and February 2025. All experimental protocols strictly adhered to the ARRIVE guidelines and were approved by the Laboratory Animal Ethics Committee of the University of South China (Approval No. USC2024XS020).

### Experimental animals

Specific pathogen-free (SPF) BALB/c mice (5–6 weeks old, with a 1:1 sex ratio) were obtained from Slake Jingda Experimental Animal Co., Ltd. (Hunan, China; Animal Production License No. SCXK(Hunan)2021-0002) and housed in the barrier facility of the Laboratory Animal Center at the University of South China. The housing conditions included controlled temperature (22 ± 1 °C), humidity (55 ± 5%), and a 12-hour light/dark cycle, with free access to sterilized feed and water.

### Strains and cells

The *Mp* reference strain M129 (ATCC 29342) was maintained at the Institute of Pathogenic Biology, Hengyang Medical College, University of South China. For experimental use, thawed stock cultures were propagated in pleuropneumonia-like organism (PPLO) broth (BD Difco) supplemented with 20% (v/v) heat-inactivated fetal bovine serum (FBS; ExCell Bio), 0.5% (w/v) glucose (Solarbio), 0.4% (w/v) yeast extract (Solarbio), and 160 U/mL penicillin G (Macklin), incubated at 37 °C.

BEAS-2B (ATCC CRL-3588) cell lines (Sunncell) were cultured in DMEM (high glucose, 4.5 g/L; Sigma-Aldrich) containing 10% (v/v) FBS and penicillin-streptomycin solution (100 U/mL penicillin, 100 ng/mL streptomycin; Epizyme), with routine subculture in a humidified 5% CO_2_ incubator at 37 °C.

### Prediction and screening of T cell epitope and B cell epitope

Immunodominant epitopes from the P1 adhesin and CARDS toxin were identified using a multi-algorithm consensus strategy. T cell epitopes were predicted by consensus of the IEDB Analysis Resource (https://www.iedb.org/, score > 0.6), and SYFPEITHI (http://www.syfpeithi.de/, score > 20), with positive hits requiring agreement in at least two of these algorithms. B cell epitope screening combined with linear epitope predictions from BepiPred 2.0 (https://services.healthtech.dtu.dk/services/BepiPred-2.0/) and ABCpred (https://webs.iiitd.edu.in/raghava/abcpred/), selecting sequences with combined probability scores > 0.5. All candidate epitopes were further evaluated for biosafety through antigenicity prediction using VaxiJen 2.0 (https://ddg-pharmfac.net/vaxijen/VaxiJen/VaxiJen.html, threshold > 0.4), allergenicity screening via AllerTOP 2.1 (https://ddg-pharmfac.net/allertop_test/, probability < 0.5 for non-allergens), and toxicity assessment with ToxinPred (https://webs.iiitd.edu.in/raghava/toxinpred2/batch.html, SVM score < 0.5 for non-toxic sequences). Sequences passing all filters were retained for vaccine development.

### MEV design and property evaluation

The MEV P1Tx construct was developed by systematically linking CD4^+^ T cell epitopes with GPGPG linkers and B cell epitopes with KK linkers. Computational analysis was conducted using ProtParam (https://web.expasy.org/protparam/) to evaluate key physicochemical properties, including molecular weight, theoretical isoelectric point, instability index, aliphatic index, and hydropathicity profile. Immunological safety was verified through antigenicity (VaxiJen 2.0), allergenicity (AllerTOP 2.1), and toxicity (ToxinPred).

### Structural evaluation of P1Tx

The secondary structure of P1Tx was predicted using the SOPMA online platform (https://npsa-pbil.ibcp.fr/cgibin/npsa_automat.pl?page=npsa_sopma.html). A three-dimensional structural model was then created with a deep learning architecture of AlphaFold 3 (https://alphafoldserver.com/). This model was thoroughly validated, including structural visualization with PyMOL 3.1.5.1, Ramachandran plot analysis using PyMod 3.0, and Z-score calculation through ProSA-web (https://prosa.services.came.sbg.ac.at/prosa.php/) to evaluate its structural reliability.

### Recombinant proteins

The DNA sequences encoding the P1Tx, P1C adhesin (residues 1125–1518), and CARDS toxin non-toxic mutant (CARDS_mu_; E269A/E271A substitutions (29, 34)) were then cloned and inserted into the *Escherichia coli* expression vector pET-28a(+) to produce recombinant plasmids. All recombinant plasmids were synthesized and sequence-verified by Sangon Biotech (Shanghai, China). The recombinant plasmid encoding CARDS toxin was generously provided by Professor Jun He of the Second Affiliated Hospital, University of South China (Hunan, China). All recombinant proteins were expressed in *E. coli* BL21(DE3) under optimized conditions as described (35): P1Tx was induced with 0.5 mM IPTG at 16 °C for 16 h for inclusion body expression, P1C with 0.5 mM IPTG at 37 °C for 5 h, while CARDS toxin and CARDS_mu_ with 0.5 mM IPTG at 16 °C for 16 h for soluble expression. Proteins were purified by Ni-NTA affinity chromatography under denaturing (P1Tx) or native (P1C, CARDS toxin, and CARDS_mu_) conditions, followed by endotoxin removal (<0.05 EU/mg) and stored at -80 °C after confirmation by Western blot with anti-His-tag antibody and quantified by BCA.

### Enzyme-linked immunosorbent assay

To quantify serum IgG and its subclasses, 96-well plates were coated with 100 μL/well of antigens (10–20 μg/mL) at 4 °C overnight, then blocked with 5% skim milk at 37 °C for 2 h. Next, 100 μL of diluted serum (1:200 for *Mp*-infected patient sera; 1:50,000 for immunized mice; 1:20 for *Mp*-infected mouse sera) was added and incubated at 37 °C for 1 h. After three PBST washes, plates were incubated with HRP-conjugated goat anti-human or mouse IgG (1:20,000), IgG1 (1:2,000), or IgG2a (1:2,000) at 37 °C for 2 h. The TMB substrate was then added at room temperature for 30 min. Reactions were quenched with 2 M H_2_SO_4_, and absorbance was measured at 450 nm (OD_450_). Samples were considered positive if the OD_450_ ratio (sample/negative control) was ≥ 2.1 (35).

Concentrations of IL-1β, TNF-α, IL-6, IL-10, and IL-17A in lung homogenate supernatants were quantified using commercial ELISA kits (Thermo Scientific™) according to the manufacturer’s instructions. Cytokine levels, expressed as pg per mg of total protein (pg/mg), were calculated based on sample OD_450_ values.

### Immunofluorescence analysis

To assess the adhesion of P1Tx to BEAS-2B cells, confluent monolayers in 24-well plates were incubated with 150 μL of P1Tx protein at concentrations of 100 μg/mL for 2 h at 37 °C. *Mp* suspension and untreated cells served as positive and negative controls, respectively. After incubation, the cells were fixed, blocked, and incubated with primary antibodies (mouse anti-His-tag antibody and rabbit anti-*Mp* monoclonal antibodies). Then, samples were incubated with Cy3-conjugated goat anti-mouse/rabbit secondary antibodies. Nuclei were counterstained with DAPI, and the slides were examined under a fluorescence microscope to evaluate the adhesion of the target protein and pathogen to the cells.

To evaluate the capacity of P1Tx antibodies to block *Mp* adhesion to BEAS-2B cells, we purified P1Tx-specific IgG (100 μg/mL in PBS) from convalescent sera of MPP patients using standardized protocols (36, 37). 50 μL of *Mp* culture (2.5 × 10^6^ CFU/mL in PPLO broth) was mixed with 50 μL of test sera (from immunized mice or purified P1Tx antibodies) and pre-incubated at 37 °C for 2 h. Negative controls included *Mp* culture without antibodies, while positive controls involved *Mp* culture with serum samples from MPP patients incubated with PBS. The pre-treated *Mp* suspensions were then added to BEAS-2B cells. After a 2-hour adhesion period at 37 °C, cells were processed for immunofluorescence staining (fixation, blocking, primary and secondary antibody incubation, DAPI counterstaining) to measure *Mp* adhesion.

### Optical microscopy analysis

To evaluate the potential vacuolating toxicity of P1Tx, BEAS-2B cells were treated with 50 μL of purified P1Tx, CARDS toxin, or CARDS_mu_ (each at 2 mg/mL) and incubated at 37 °C with 5% CO_2_. Cell morphology and vacuolation were monitored every 4 hours using microscopy. After 40 hours, images were taken to quantify vacuole formation. To assess toxin activity neutralization, CARDS toxin (2 mg/mL) was pre-incubated for 2 hours at 37 °C with serum antibodies (either a 1:100 dilution of immunized mouse serum or 100 μg/mL P1Tx antibodies purified from serum samples of MPP patients). This mixture was then added to BEAS-2B cells. Vacuole formation was quantified using the same methods.

### Mouse studies

Mice were randomly assigned to four groups (n = 20 per group) and immunized subcutaneously at multiple sites (neck and back) with 50 μg of P1Tx, P1C protein, CARDSmu, or PBS emulsified in an equal volume of Freund’s adjuvant. Immunizations were administered three times at 2-week intervals, using complete Freund’s adjuvant for the primary dose and incomplete adjuvant for boosters. The sample size was chosen based on practical constraints and common practices in similar studies.

Blood samples were collected via tail vein incision on days 0, 14, and 28 post-immunization. Sera were separated and stored for subsequent analysis. On day 42, mice (10 per group) were euthanized by gradual displacement (30% chamber volume per minute) with compressed CO_2_. Respiratory arrest (>30 s apnea) was confirmed before performing cardiac puncture, followed immediately by cervical dislocation. Spleens were harvested after confirmation of death.

The remaining mice (10 per group) were challenged intranasally under isoflurane anesthesia with 1×10^8^ CFU of *Mp* M129 strain in 50 μL sterile PBS. Four days post-challenge, animals were euthanized using the aforementioned CO_2_ protocol, and both spleen and lung tissues were collected for immune protection assessment.

### Flow cytometry analysis

To detect intracellular cytokines in spleen cells, single-cell suspensions from immunized mice were stimulated with PMA (50 ng/mL), ionomycin (1 μg/mL), and Brefeldin A (1 μg/mL) for 5 hours. After Fc blocking, cells were surface-stained with anti-CD3, CD4, and CD8 antibodies, followed by the addition of Live/Dead viability dye to exclude dead cells. After fixation and permeabilization, intracellular staining was performed using anti-IFN-γ and anti-IL-4 antibodies. For pulmonary cells isolated after the *Mp* challenge, the lung tissue was processed as described (38): the homogenized tissue was centrifuged (1200×g, 5 min), resuspended in 35% Percoll, and then centrifuged again (700×g, 15 min). The cells were treated with ACK lysis buffer to lyse erythrocytes, then washed and resuspended in PBS. Lung cells underwent the same intracellular cytokine detection protocol as spleen cells. FMO controls were included for all multicolor panels to establish gating boundaries.

Splenic lymphocyte subsets were characterized using polychromatic flow cytometry (FCM). After blocking Fc receptors, T cell subsets were identified by combining Live/Dead viability dye with antibodies against CD3, CD4, CD8, CD44, and CD62L. B cell subsets were distinguished using Live/Dead dye along with antibodies against CD19, CD45R (B220), CD38, FAS, and GL7.

To evaluate antibody-mediated adhesion inhibition, FITC-labeled Mp was pre-incubated with serum for 1 hour. Unbound antibodies were removed through centrifugation (12,000×g, 10 min), and the mixture was then incubated with BEAS-2B cells for 2 h (39, 40).

All data were collected using a BD FACSCanto II flow cytometer and analyzed with FlowJo 10.8. The gating strategies were shown in **Supplementary File 1**.

### *Mp* loading in lung tissue

100 μL of homogenate supernatant was uniformly applied to PPLO agar plates (six-well format) and incubated at 37 °C for 10 days as described (41, 42). Colonies were enumerated under an optical microscope (×100 magnification), and colony-forming units (CFU) were calculated using the formula:


$$\text{CFU}/\text{mL}=\frac{\text{Total colonies in }10\text{ fields of view}}{10}\times \frac{\text{Agar plate area}}{\text{Single field area}}\times \text{Dilution factor}\times (1000\text{µL}/\text{Plating volume})$$

Each mouse was independently tested in triplicate.

### Pathological injury to lung tissue

The right lower lobes from infected mice were fixed in 4% paraformaldehyde for 24 h. Paraffin embedding, sectioning, and hematoxylin-eosin (HE) staining were performed by Hunan Aifang Biotechnology Co., Ltd. Pathological scoring was conducted double-blind using criteria adapted from previous studies (43, 44).

### Statistics and reproducibility

Statistical analysis was performed using one-way or two-way ANOVA, followed by the Tukey or Dunnett *post hoc* test for multiple comparisons among groups. Results are expressed as mean ± standard deviation (SD). A significance threshold of *P* < 0.05 was applied. Data analysis was conducted using GraphPad Prism v10.1.2.

## Results

### Epitope screening, vaccine construction, and recombinant expression

Immunoinformatics screening identified CD4+ T cell epitopes from Mp adhesion protein P1 and CARDS toxin using MHC-II binding prediction tools. Dominant epitopes were selected based on consensus thresholds: an IEDB score greater than 0.6, and a SYFPEITHI score greater than 20. B cell epitopes were predicted by analyzing IEDB and ABCpred datasets (**Supplementary Tables S1**–**S4**). High-antigenicity (VaxiJen 2.0 >0.4), non-allergenic (AllerTop 2.1), and non-toxic (ToxinPred) epitopes were fused via GPGPG/KK linkers to construct the 263-amino acid chimeric vaccine, P1Tx (**Figure 1A**). ElliPro predicted 5 linear B cell epitopes (7–53 residues) and 12 conformational B cell epitopes (4–29 residues) within P1Tx (**Supplementary Tables S5**, **S6**; **Supplementary Figure S1**). Conformational epitope scores ranged from 0.538 to 0.908, indicating high conformational flexibility.

**Figure 1:**
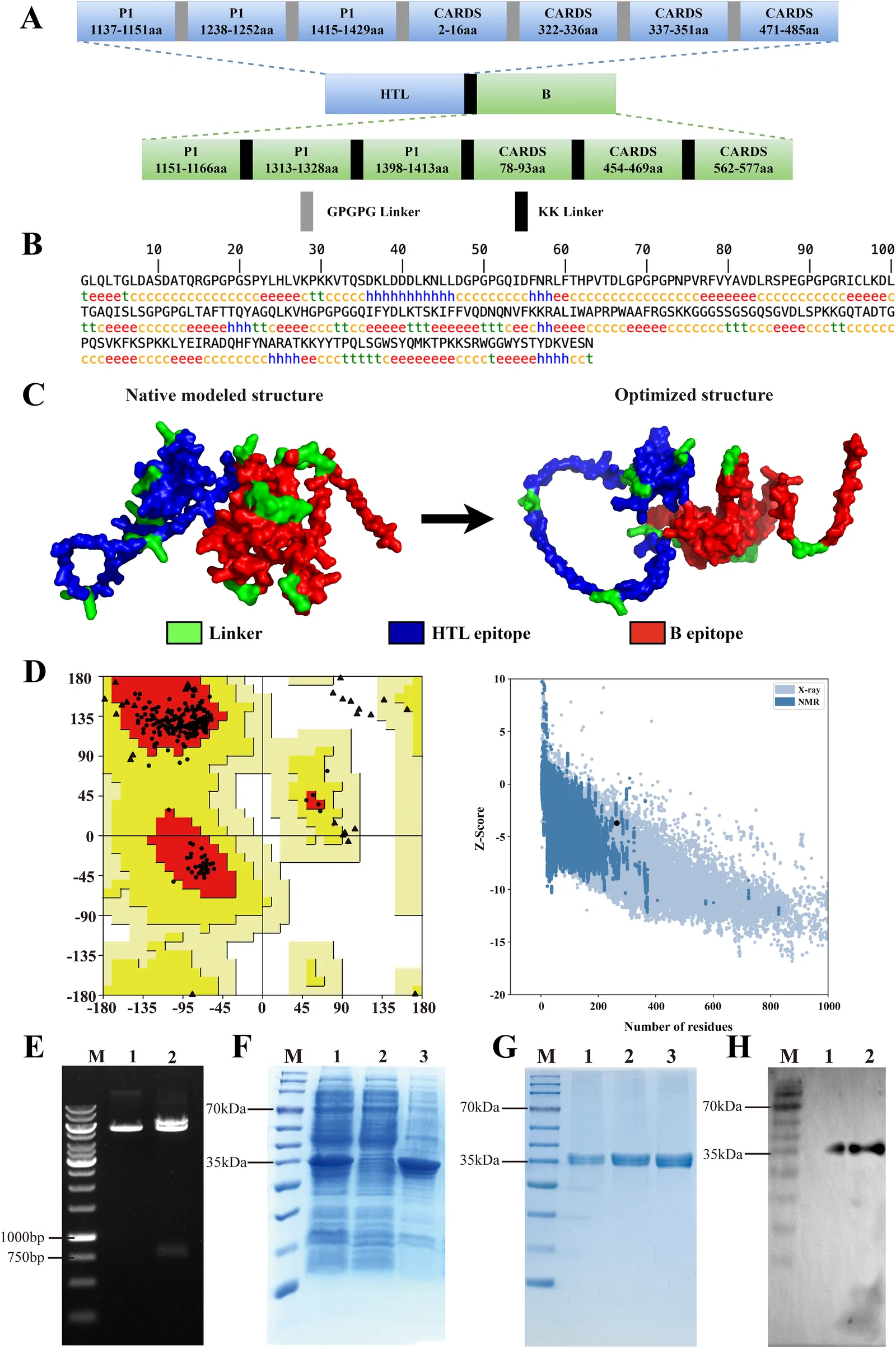
Design, construction, and expression of P1Tx. **(A)** P1Tx amino acid sequence design. **(B)** Predicted secondary structure of P1Tx. Colors: blue, α-helix; green, β-turn; red, extended strand; yellow, random coil. **(C)** P1Tx 3D structure modeling and refinement. Left: AlphaFold3 initial structure; Right: Galaxy Refine refined structure. **(D)** Ramachandran plot and Z-score. The plot shows 95% residues in favored, 4.0% in allowed, 0.5% in outlier regions, and 0% in disallowed regions. Model Z-score: -3.69. **(E)** BamHI/XhoI double digestion of pET-28a(+)-P1Tx plasmid. M: DNA marker; Lane 1: Digestion products of pET-28a plasmid; Lane 2: Digestion products of pET-28a(+)-P1Tx plasmid. **(F)** SDS-PAGE analysis of P1Tx expression in *E. coli* BL21(DE3). Lane 1: Whole-cell lysate; Lane 2: Soluble fraction; Lane 3: Insoluble fraction; M: Protein marker. **(G)** Purification of P1Tx. Lanes 1-3: Purification steps. **(H)** Western blot detection of P1Tx expression. Lanes 1-2: P1Tx; M: Protein marker. The full images of **(E–H)** were shown in **Supplementary File 2**.

P1Tx exhibited an expected molecular weight of 28 kDa, hydrophilicity (GRAVY = -0.701), and absence of toxicity or allergenicity (**Supplementary Table S7**). Secondary structure analysis by SOPMA showed 10.7% α-helices, 10.65% β-turns, 32.32% extended strands, and 46.77% random coils **Figure 1B**). The secondary-structure prediction reveals limited fractions of α-helices and β-turns, together with a large proportion of random coils. In contrast, random coils may provide functional flexibility. The tertiary structure, modeled with AlphaFold3 and refined using Galaxy Refine, exhibited 95% of residues in the favored regions of the Ramachandran plot, with no residues in disallowed regions, and a ProSAweb Z-score (-3.69) that falls within the range of native proteins of similar size, supporting the predicted structural stability of the P1Tx model in silico (**Figures 1C, D**).

The optimized gene (789 bp) was cloned into pET-28a(+) (**Figure 1E**) and expressed in E. coli BL21(DE3), producing P1Tx inclusion bodies (**Figure 1F**). Purification through Ni-NTA affinity chromatography, followed by urea denaturation and dialysis for refolding, showed a clear ~28 kDa band on SDS-PAGE (**Figure 1G**), matching the predicted molecular weight. The identity of recombinant P1Tx was confirmed by Western blot using an anti-His-tag antibody (**Figure 1H**). Parallel expressions of P1C, CARDS toxin, and CARDSmu are shown in **Supplementary Figure S2**.

### Recombinant P1Tx demonstrates strong immunogenicity and adhesion *in vitro* without vacuolating activity, indicating promising vaccine prospects

The immunoreactivity of recombinant P1Tx with serum IgG and IgM from *Mp*-infected patients was evaluated using indirect ELISA (**Figure 2A**). P1Tx showed significantly stronger binding to both IgG and IgM compared to PBS controls (*P* < 0.05), although it exhibited weaker IgG affinity than *Mp*-positive and P1C controls (*P* < 0.001). IgM binding was also significantly lower than that of *Mp*, P1C, and CARDS_mu_ controls (*P* < 0.05).

**Figure 2:**
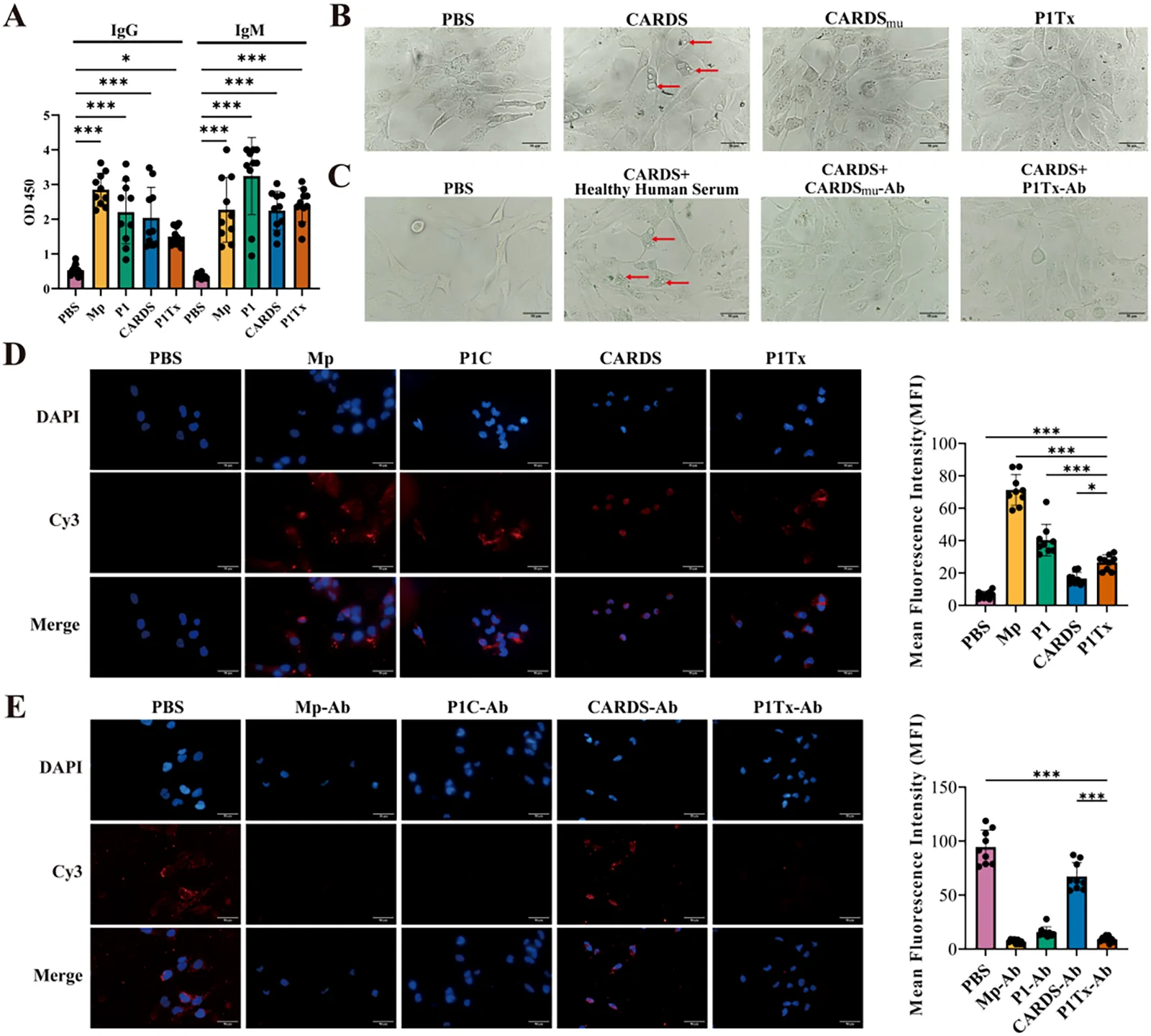
Immunoreactivity, vacuolation, and adhesion of P1Tx. **(A)** ELISA analysis of immunoreactivity between the P1Tx protein and MPP patient serum. **(B)** Vacuolating toxicity analysis of P1Tx. Red arrows indicate representative vacuoles. **(C)** Neutralization of CARDS toxin vacuolating activity by purified P1Tx antibodies. **(D)** IFA of P1Tx adhesion to BEAS-2B cells. **(E)** IFA of inhibitory effects of P1Tx-specific antibodies. Images **(B–E)** were captured at 400× magnification (scale bar = 50 μm). Statistical analysis was performed using one-way ANOVA, followed by the Tukey *post hoc* test for multiple comparisons among groups. *P < 0.05, ***P < 0.001.

Next, vacuolating effects were explored due to P1Tx’s partial sequence homology with vacuole-inducing CARDS toxin domain III. Microscopy showed extensive vacuolation in BEAS-2B cells treated with CARDS toxin. In contrast, cells treated with P1Tx or CARDS_mu_ maintained normal morphology (**Figure 2B**). Quantitative analysis indicated significantly higher vacuole formation in the groups treated with CARDS toxin compared to P1Tx and CARDS_mu_ (*P* < 0.001) (**Supplementary Figure S4**). Compared to the control, both CARDS toxin- and P1Tx-specific antibodies resulted in a marked reduction in vacuole formation (*P* < 0.001) (**Figure 2C**; **Supplementary Figures S3**, **S4**).

Additionally, P1Tx adhesion to BEAS-2B cells was evaluated by IFA. Preliminary experiments demonstrated dose-dependent adhesion for P1C, CARDS toxin, and P1Tx, with optimal binding at a concentration of 100 μg/mL. Mean-fluorescence-intensity (MFI) quantification revealed that P1Tx conferred markedly stronger adhesion than either PBS controls or CARDS toxin (*P* < 0.05). However, it was weaker than *Mp*-positive controls and P1C (*P* < 0.001) (**Figure 2D**). Furthermore, antibodies specific to P1Tx, P1C, or *Mp* all significantly reduced *Mp* adhesion compared to CARDS toxin-specific antibodies (*P* < 0.001) (**Figure 2E**).

### P1Tx induces strong humoral immune responses in mice

To evaluate the immune responses induced by P1Tx immunization, mouse immunization models were established as shown in **Figure 3A**. FCM analysis of spleens 14 days after the final immunization (**Figures 3G, H**) showed a significantly higher proportion of germinal center (GC) B cells in the P1Tx group compared to both P1C and PBS control groups (*P* < 0.001), indicating effective GC reaction activation. Conversely, the P1Tx group had significantly lower MBC levels than PBS, P1C, and CARDS_mu_ groups (*P* < 0.01), potentially due to transient GC B cell expansion and delayed MBC development.

**Figure 3:**
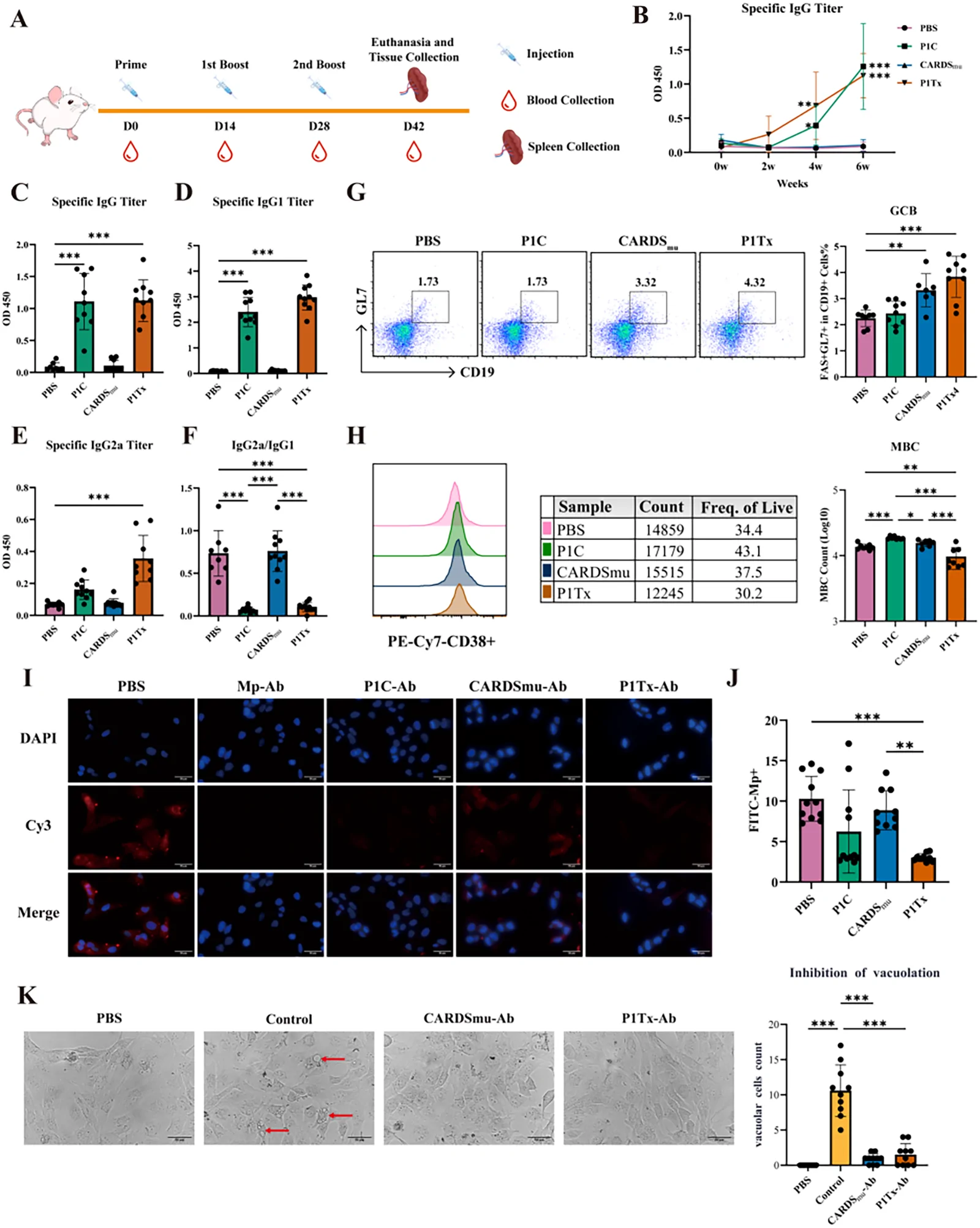
Humoral immune responses induced by P1Tx. **(A)** Immunization strategy schedule in the mouse model. **(B)** Serum-specific IgG levels over time. **(C–F)** Week 6 titers of specific IgG, IgG1, IgG2a, and the IgG2a/IgG1 ratio. **(G, H)** FCM quantified splenic GC B cells and MBC cells. **(I)** IFA-based adhesion inhibition assay using immune serum. **(J)** Flow cytometric quantification of immune serum-mediated adhesion inhibition. **(K)** Neutralization of vacuolating toxin activity by immune serum and statistical quantification. Images **(I, K)** were captured at 400× magnification (scale bar = 50 μm). Statistical analysis of **(B)** was performed using two-way ANOVA, followed by the Dunnett *post hoc* test for multiple comparisons among groups. Statistical analysis of the others was performed using one-way ANOVA, followed by the Tukey *post hoc* test for multiple comparisons among groups. * *P* < 0.05, **P < 0.01, ***P < 0.001.

Serum IgG antibody titers in the P1Tx group steadily increased over time (**Figure 3B**). By week 4 after the initial immunization, IgG levels were significantly higher than those in the PBS and CARDS_mu_ groups (*P* < 0.05), and this difference grew more pronounced by week 6 (*P* < 0.001) (**Figure 3C**). Serum subclass analysis at 2 weeks after the final immunization (**Figures 3D, E**) showed that P1Tx induced notably higher IgG1 and IgG2a titers (*P* < 0.001), while producing a significantly lower IgG2a/IgG1 ratio compared to PBS and CARDS_mu_ groups (*P* < 0.001), reaching levels similar to the P1C group (**Figure 3F**). IFA results indicated that P1Tx immune serum markedly decreased the MFI of *Mp* adhesion to BEAS-2B cells (*P* < 0.001) (**Figure 3I**; **Supplementary Figure S5**). This reduction was confirmed by FCM, which measured FITC-labeled *Mp* adhesion rates (*P* < 0.01) (**Figure 3J**; **Supplementary Figure S5**). Additionally, pretreatment with immune serum from P1Tx or CARDS_mu_ significantly reduced CARDS toxin-induced vacuolated cells (*P* < 0.001) (**Figure 3K**; **Supplementary Figure S6**), demonstrating that both sera neutralize the cytotoxic effects of CARDS toxin. These findings suggest that P1Tx elicits a robust humoral immune response, resulting in the production of neutralizing antibodies.

### P1Tx activates cellular immune responses

In addition to eliciting strong humoral immune responses, P1Tx immunization also triggered potent cellular immune activation. FCM analysis of splenic T cell subsets at 14 days after the final immunization (**Figures 4A–D**) showed that the P1Tx group had significantly lower frequencies of both CD4^+^ naive T cells (TN, CD44^-^CD62L^+^) and CD8^+^ TN populations compared to the PBS control group (*P* < 0.001), with the CD4^+^ TN subset decreasing further relative to the P1C subunit vaccine control (*P* < 0.01). These results align with vaccine-induced T cell activation and subsequent clonal expansion. Regarding effector T cells (Teff, CD44^+^CD62L^-^), the P1Tx group exhibited notably higher proportions of CD4^+^ Teff cells compared to both PBS and P1C groups (*P* < 0.01), along with significantly increased CD8^+^ Teff levels relative to the PBS control (*P* < 0.05). Additionally, both CD4^+^ and CD8^+^ central memory T cell (TCM, CD44^+^CD62L^+^) populations were considerably increased in the P1Tx group compared to both PBS and P1C controls (*P* < 0.05).

**Figure 4:**
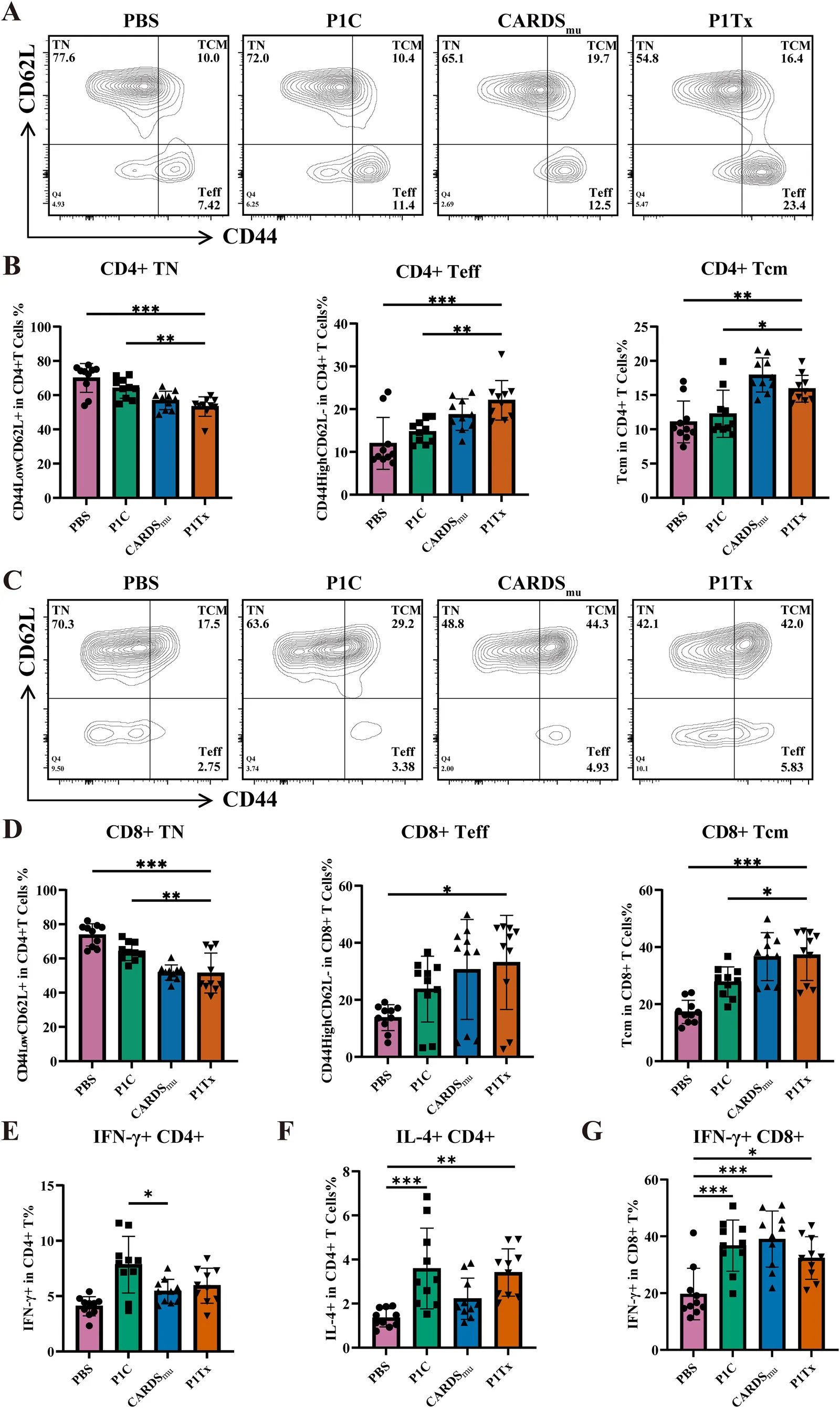
Cellular immune responses induced by P1Tx. **(A)** Representative flow data of CD4+ T cell subsets. **(B)** Statistical analysis of the proportions of CD4+ TN, Teff, and TCM within the CD4+ T cell population. **(C)** Representative flow data of CD8+ T cell subsets. **(D)** Statistical analysis of the proportions of CD8+ TN, Teff, and TCM within the CD8+ T cell population. **(E, F)** The profiles and statistical analysis of CD4+ T cells that expressed IFN-γ and IL-4. **(G)** The profiles and statistical analysis of CD8+ T cells that expressed IFN-γ. Statistical analysis was performed using one-way ANOVA, followed by the Tukey *post hoc* test for multiple comparisons among groups. * *P* < 0.05, ** *P* < 0.01, ***P < 0.001.

Intracellular cytokine detection (**Figures 4E–G**; **Supplementary Figure S7**) revealed that P1Tx specifically promoted the expansion of Th2 cells (CD4^+^IL-4^+^) and cytotoxic T lymphocytes (CTL; CD8^+^IFN-γ^+^). Notably, the proportion of IL-4^+^ CD4^+^ T cells did not differ significantly between the P1Tx and P1C groups; the Th2 bias of the P1Tx-induced response is instead supported by its IgG1-dominant subclass profile (**Figure 3F**). Overall, these results indicate that P1Tx primarily enhances humoral and cytotoxic responses through a Th2/CTL-biased pathway.

### P1Tx vaccination enhances pathogen-specific immunity and alleviates pulmonary inflammatory pathology in *Mp*-infected mice

The *Mp* infection protocol for immunized mice is outlined in **Figure 5A**. Serum levels of *Mp*-specific IgG antibodies in the P1Tx-immunized group were significantly higher than those in the PBS, P1C, and CARDS_mu_ groups (*P* < 0.001) (**Figure 5B**). Intracellular cytokines IFN-γ and IL-4 in lung T cells on day 4 post-infection (**Figures 5C–E**; **Supplementary Figure S8**) showed that P1Tx immunization significantly boosted the activation of Th1, Th2, and CTL cells compared to all three control groups (*P* < 0.001).

**Figure 5:**
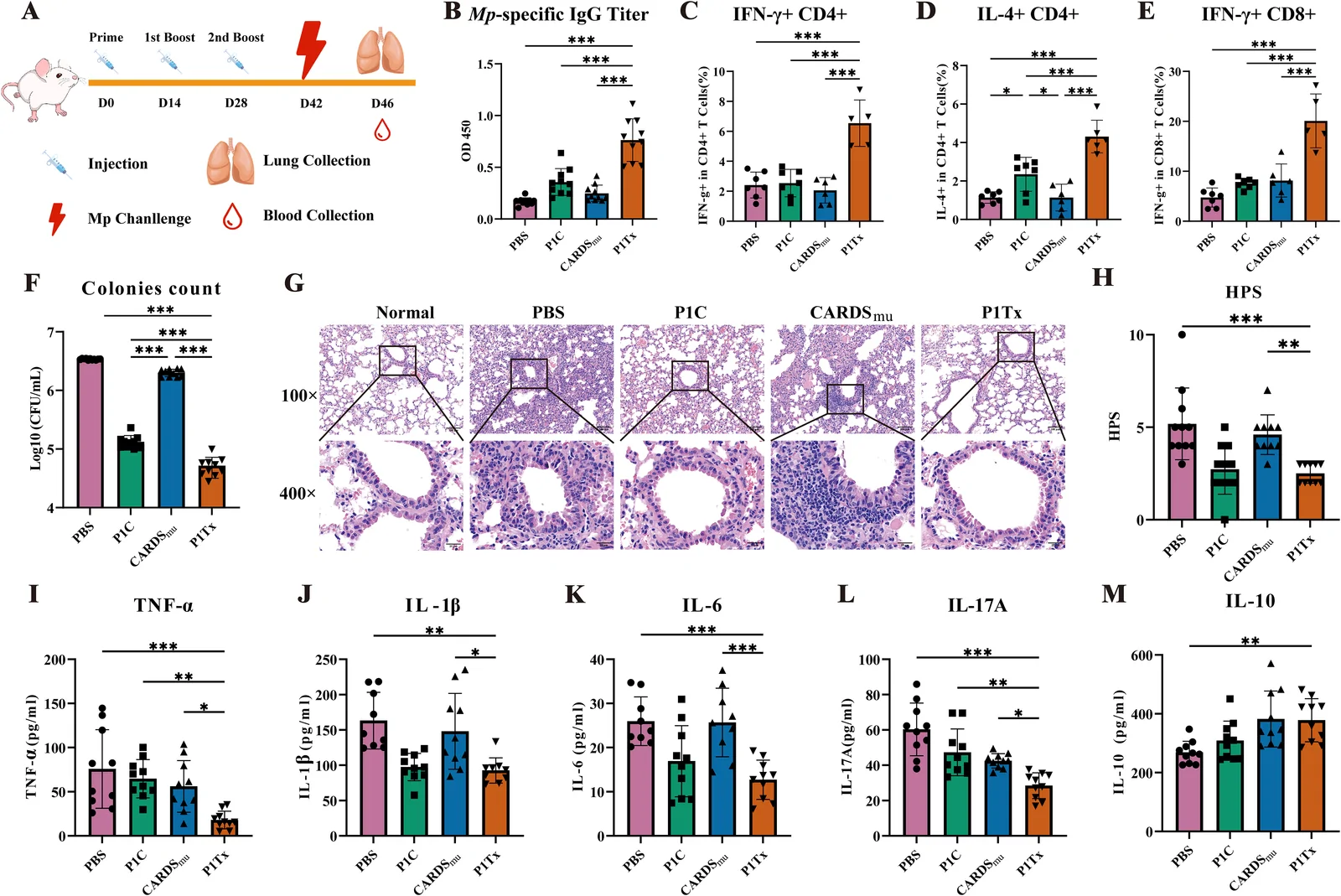
P1Tx enhances specific immune response and mitigates lung inflammatory pathology in mice infected with *Mp*. **(A)** Schematic diagram of the establishment of the BALB/c mouse immune protection model. **(B)** Detection of *Mp*-specific IgG in mouse serum on day 4 post-infection by ELISA. **(C, D)** The profiles and statistical analysis of CD4^+^ T cells that expressed IFN-γ and IL-4. **(E)** The profiles and statistical analysis of CD8^+^ T cells that expressed IFN-γ. **(F)** The colony counts of *Mp* colonies cultured from the supernatant of the mouse pulmonary homogenate on day 4 post-infection. **(G)** Representative images (×100 and ×400) of mouse lung sections on day 4 post-infection. **(H)** Histological pathological score of lung tissue sections. **(I–M)** ELISA assay of cytokines in mouse pulmonary homogenate supernatant on day 4 post-infection. Statistical analysis was performed using one-way ANOVA, followed by the Tukey *post hoc* test for multiple comparisons among groups. *P < 0.05, **P < 0.01, ***P < 0.001.

On day 4 after infection, the P1Tx-immunized group showed a significantly lower *Mp* load in lung tissues compared to the PBS and CARDS_mu_ groups, with CFU counts reduced to just 1.5% of the levels in the PBS group (*P* < 0.001). However, there was no significant difference when compared to the P1C group (**Figure 5F**; **Supplementary Figure S9**). Lung histopathology indicated minimal inflammatory infiltration, rare exudation, preserved alveolar structures, and a lower histological pathological score (HPS) in the P1Tx group relative to PBS and CARDS_mu_ controls (*P* < 0.01), with results similar to the P1C group (**Figures 5G, H**). Additionally, immunization with P1Tx and P1C significantly reduced the levels of pro-inflammatory cytokines (TNF-α, IL-1β, IL-6, and IL-17A) compared to PBS and CARDS_mu_ controls, while increasing the levels of the anti-inflammatory cytokine IL-10. Importantly, TNF-α and IL-17A levels were also lower in the P1Tx group than in the P1C group (*P* < 0.01) (**Figures 5I–M**). Overall, these results indicate that P1Tx aids *Mp* clearance, lessens inflammatory lung injury, and provides immunoprotection comparable to that of the P1C protein vaccine in infected mice.

## Discussion

The present study demonstrates that the MEV P1Tx, designed to target both the P1 adhesin and the CARDS toxin of *Mp*, elicits robust humoral and cellular immunity and confers significant protection in a murine model. P1Tx induced high-titer IgG, dominated by the IgG1 subclass, indicative of a Th2-biased response that is frequently associated with mucosal protection (45–48). The expansion of GC B cells and accumulation of TCM further suggest that P1Tx establishes long-lived immunological memory (49). Sera from P1Tx-immunized mice not only blocked *Mp* adhesion to human bronchial epithelial cells but also neutralized CARDS-toxin-mediated vacuolation, confirming that the selected epitopes cover functionally critical receptor-binding and toxin domains. Dual-targeting of adhesin and toxin has previously improved efficacy in *S. pneumoniae* and *Bordetella pertussis* vaccines (50, 51), and our data extend this concept to *Mp*.

Compared with P1C, P1Tx showed significantly weaker adhesion to BEAS-2B cells. This likely reflects the multi-epitope design, which incorporates only selected P1 epitopes assembled via peptide linkers and may introduce conformational constraints or epitope masking that reduce receptor-binding affinity. While we acknowledge this as a limitation, our findings indicate that the reduced intrinsic adhesion of the recombinant antigen does not impair vaccine efficacy. The selected epitopes evidently retain sufficient immunogenicity to elicit protective antibodies, and the inclusion of CARDS toxin sequences appeared to enhance cellular priming beyond that achieved by P1C alone, as reflected by the superior expansion of CD4+ and CD8+ effector and memory T cell populations. These robust humoral and cellular responses likely compensate for the reduced direct binding of P1Tx to host cells.

A legitimate concern is the high sequence diversity of P1, especially within the RepMP4 and RepMP2/3 repeats, which drive strain-specific variation (52). To address this, we focused on epitopes within the C-terminal region (aa 1125–1518). This segment contains the majority of conserved adhesion motifs, while showing the lowest sequence variability across strains (52, 53). In addition, CARDS-toxin-derived epitopes are virtually invariant across sequenced *Mp* isolates (54), offering a second layer of protection that is independent of P1 variability. Future studies will be necessary to formally demonstrate cross-strain efficacy, using methods such as passive-transfer assays or challenge experiments with a genetically diverse panel of clinical isolates.

Neutralizing antibodies frequently recognize discontinuous, conformational epitopes (55). Although P1Tx was constructed using in silico-predicted linear sequences, structural modeling indicates that 55.5% of its residues participate in conformational epitopes (ElliPro score>0.5; the percentage was calculated by dividing the number of residues located within ElliPro-predicted conformational epitopes by the total 263 residues of P1Tx; **Supplementary Table S6**; **Supplementary Figure S1**). The AlphaFold3-predicted structure shows all residues in favored Ramachandran regions and a ProSA Z-score of -3.69, consistent with a natively folded protein and suggesting that most epitopes are presented in their native three-dimensional context (56). Quantitative ElliPro analysis revealed the following B cell epitope contributions: linear- P1C 44.7%, CARDSmu 53.3%, P1Tx 52.8%; conformational-P1C 44.9%, CARDSmu 50.4%, P1Tx 55.5%. Importantly, both P1Tx-specific antibodies purified from MPP patient serum and immune sera against P1Tx significantly reduced *Mp* adhesion and attenuated CARDS toxin-induced vacuolation. These functional outcomes confirm that the in silico design successfully recapitulates conformation-dependent neutralizing structures.

This study provides preliminary insights into P1Tx, though several limitations remain. First, the epitopes were selected in silico and still require experimental validation through human T cell responses or MHC-binding assays (57). Second, immune responses were assessed only at 14 days after the final immunization, and protection was evaluated merely at 4 days post-challenge. Thus, the longitudinal antibody kinetics, duration of immunity, and potential of the vaccine to prevent CARDS toxin-driven chronic inflammation remain unclear (58, 59). Third, the pronounced Th2 bias observed may be suboptimal for clearing intracellular bacteria. Future studies using Th1-skewing adjuvants such as GLA-SE or CpG-1018 could clarify whether a more balanced immune response enhances bacterial clearance (60). Fourth, mucosal immune parameters (e.g., BALF IgA and respiratory tract resident memory T cells) were not assessed because a subcutaneous immunization route was employed. While robust systemic humoral and cellular responses conferred protection against intranasal challenge, future studies incorporating mucosal sampling are warranted to determine whether P1Tx elicits local immune responses at the site of *Mp* entry that contribute to early pathogen containment.

In summary, P1Tx delivers the functional benefits of both P1 and CARDS-toxin vaccines in a single, easily expressed polypeptide. By integrating conserved epitopes that generate adhesion-blocking and toxin-neutralizing antibodies, P1Tx represents a promising next-generation candidate against *Mp* respiratory disease.

## Conclusion

In this work, we developed P1Tx, a chimeric multi-epitope vaccine that fuses conserved epitopes of the P1 adhesin and CARDS toxin of *Mp*. Three subcutaneous doses in BALB/c mice elicited high-titer, adhesion-blocking and toxin-neutralizing antibodies, expanded GC B cells and long-lived CD4^+^/CD8^+^ memory T cells. After intranasal challenge with *Mp* M129 strain, P1Tx accelerated bacterial clearance and markedly reduced infection-associated immunopathology within four days. While human immunogenicity, durability, and adjuvant optimization remain to be tested, P1Tx integrates the protective functions of two key virulence factors into a single, readily expressed polypeptide, offering an advanced candidate for a long-needed vaccine against MPP.

## Data Availability

The data that support the findings of this study are openly available in Science Data Bank: https://doi.org/10.57760/sciencedb.27991, reference 61 (61).

## References

[1] Kingston JR Chanock RM Mufson MA Hellman LP James WD Fox HH . Eaton agent pneumonia. JAMA. (1961) 176:118–23. doi: 10.1001/jama.1961.03040150034009 13756133

[2] Yiwen C Yueyue W Lianmei Q Cuiming Z Xiaoxing Y . Infection strategies of mycoplasmas: Unraveling the panoply of virulence factors. Virulence. (2021) 12:788–817. doi: 10.1080/21505594.2021.1889813 33704021 PMC7954426

[3] He J Liu M Ye Z Tan T Liu X You X . Insights into the pathogenesis of Mycoplasma pneumoniae (Review). Mol Med Rep. (2016) 14:4030–6. doi: 10.3892/mmr.2017.8324 27667580 PMC5101875

[4] Xiu F Li X Liu L Xi Y Yi X Li Y . Mycoplasma invasion into host cells: An integrated model of infection strategy. Mol Microbiol. (2024) 121:814–30. doi: 10.1111/mmi.15232 38293733

[5] Butpech T Tovichien P . Mycoplasma pneumoniae pneumonia in children. World J Clin cases. (2025) 13:99149. doi: 10.12998/wjcc.v13.i5.99149 39959768 PMC11606359

[6] Bradley JS Byington CL Shah SS Alverson B Carter ER Harrison C . The management of community-acquired pneumonia in infants and children older than 3 months of age: clinical practice guidelines by the Pediatric Infectious Diseases Society and the Infectious Diseases Society of America. Clin Infect Dis. (2011) 53:e25–76. doi: 10.1093/cid/cir531 21880587 PMC7107838

[7] Jain S Williams DJ Arnold SR Ampofo K Bramley AM Reed C . Community-acquired pneumonia requiring hospitalization among U.S. children. N Engl J Med. (2015) 372:835–45. doi: 10.1056/nejmoa1405870 25714161 PMC4697461

[8] Meyer Sauteur PM Beeton ML . Mycoplasma pneumoniae: delayed re-emergence after COVID-19 pandemic restrictions. Lancet Microbe. (2024) 5:e100–1. doi: 10.1016/s2666-5247(23)00344-0 38008103

[9] Raghuram A Furmanek S Chandler T Rashid S Mattingly W Ramirez J . Description of a current outbreak of Mycoplasma pneumoniae in the United States. Pathogens. (2025) 14:60. doi: 10.3390/pathogens14010060 39861021 PMC11768315

[10] Kim K Jung S Kim M Park S Yang HJ Lee E . Global trends in the proportion of macrolide-resistant Mycoplasma pneumoniae infections: A systematic review and meta-analysis. JAMA Netw Open. (2022) 5:e2220949. doi: 10.1001/jamanetworkopen.2022.20949 PMC9274321 35816304

[11] Wang YS Zhou YL Bai GN Li SX Xu D Chen LN . Expert consensus on the diagnosis and treatment of macrolide-resistant Mycoplasma pneumoniae pneumonia in children. World J Pediatr. (2024) 20:901–14. doi: 10.1007/s12519-024-00831-0 39143259 PMC11422262

[12] Ding G Zhang X Vinturache A van Rossum AMC Yin Y Zhang Y . Challenges in the treatment of pediatric Mycoplasma pneumoniae pneumonia. Eur J Pediatr. (2024) 183:3001–11. doi: 10.1007/s00431-024-05519-1 38634891

[13] Tsai TA Tsai CK Kuo KC Yu HR . Rational stepwise approach for Mycoplasma pneumoniae pneumonia in children. J Microbiol Immunol Infect. (2021) 54:557–65. doi: 10.1016/j.jmii.2020.10.002 33268306

[14] Jiang Z Li S Zhu C Zhou R Leung PHM . Mycoplasma pneumoniae infections: Pathogenesis and vaccine development. Pathogens. (2021) 10:119. doi: 10.3390/pathogens10020119 33503845 PMC7911756

[15] Jiang Z Zhou R Leung PHM Deng Z Li S . An attenuated multiple genetic mutant of Mycoplasma pneumoniae imparts good immuno-protection against M. pneumoniae pneumonia in BALB/c mice. Microb Pathogen. (2022) 165:105463. doi: 10.1016/j.micpath.2022.105463 35240287

[16] Yu L Chen C He G Meng Y Long H He J . Oral vaccination with live Mycoplasma pneumoniae elicits a respiratory protective immunity in a murine model. Vaccine. (2025) 61:127460. doi: 10.1016/j.vaccine.2025.127460 40609261

[17] Xu N Fan L Li L Guo Y . Exploring the pathogenicity of Mycoplasma pneumoniae: Focus on community-acquired respiratory distress syndrome toxins. Microb Pathogen. (2024) 195:106865. doi: 10.1016/j.micpath.2024.106865 39153578

[18] Vizarraga D Kawamoto A Marcos-Silva M Martín J Makino F Miyata T . Dynamics of the adhesion complex of the human pathogens Mycoplasma pneumoniae and Mycoplasma genitalium. PloS Pathog. (2025) 21:e1012973. doi: 10.1371/journal.ppat.1012973 40153444 PMC11984735

[19] Zeng Q Sun P Li W Tang Y Hu Y Zhou J . Protective immunity induced by a novel P1 adhesin C-terminal anchored mRNA vaccine against Mycoplasma pneumoniae infection in BALB/c mice. Microbiol Spectr. (2025) 13:e0214024. doi: 10.1128/spectrum.02140-24 39831768 PMC11878037

[20] Jacobs E Pilatschek A Gerstenecker B Oberle K Bredt W . Immunodominant epitopes of the adhesin of Mycoplasma pneumoniae. J Clin Microbiol. (1990) 28:1194–7. doi: 10.1128/jcm.28.6.1194-1197.1990 1696281 PMC267904

[21] Miyata M Hamaguchi T . Integrated information and prospects for gliding mechanism of the pathogenic bacterium Mycoplasma pneumoniae. Front Microbiol. (2016) 7:960. doi: 10.3389/fmicb.2016.00960 27446003 PMC4923136

[22] Hsieh YC Li SW Chen YY Kuo CC Chen YC Chang IY . Global genome diversity and recombination in Mycoplasma pneumoniae. Emerging Infect Dis. (2022) 28:111–7. doi: 10.3201/eid2801.210497 34932441 PMC8714221

[23] Chen C Yong Q Jun G Ying P Suqin L Jiameng L . Designing, expression and immunological characterization of a chimeric protein of Mycoplasma pneumoniae. Protein Pept Lett. (2016) 23:592–6. doi: 10.2174/0929866523666160502155414 27137129

[24] Fan L Li D Zhang L Hao C Sun H Shao X . Pediatric clinical features of Mycoplasma pneumoniae infection are associated with bacterial P1 genotype. Exp Ther Med. (2017) 14:1892–8. doi: 10.3892/etm.2017.4721 28962100 PMC5609089

[25] Sun H Xue G Yan C Li S Zhao H Feng Y . Changes in molecular characteristics of Mycoplasma pneumoniae in clinical specimens from children in Beijing between 2003 and 2015. PloS One. (2017) 12:e0170253. doi: 10.1371/journal.pone.0170253 28107399 PMC5249184

[26] He J Liu M Ye Z Tan T Liu X You X . Insights into the pathogenesis of Mycoplasma pneumoniae (Review) Corrigendum in /10.3892/mmr.2017.8324. Mol Med Rep. (2016) 14:4030–6. doi: 10.3892/mmr.2016.5765 27667580 PMC5101875

[27] Krishnan M Kannan TR Baseman JB . Mycoplasma pneumoniae CARDS toxin is internalized via clathrin-mediated endocytosis. PloS One. (2013) 8:e62706. doi: 10.1371/journal.pone.0062706 23667510 PMC3647021

[28] Kannan TR Baseman JB . ADP-ribosylating and vacuolating cytotoxin of *Mycoplasma pneumoniae* represents unique virulence determinant among bacterial pathogens. Proc Natl Acad Sci. (2006) 103:6724–9. doi: 10.1073/pnas.0510644103 16617115 PMC1458948

[29] Yoshikawa E Tamiya S Inoue Y Suzuki K Yoshioka Y . Vaccine using community-acquired respiratory distress syndrome toxin as an antigen against Mycoplasma pneumoniae in mice. Biochem Biophys Res Commun. (2022) 594:81–7. doi: 10.1016/j.bbrc.2022.01.058 35078111

[30] Fan X Li X Wan K Zhao X Deng Y Chen Z . Construction and immunogenicity of a T cell epitope-based subunit vaccine candidate against Mycobacterium tuberculosis. Vaccine. (2021) 39:6860–5. doi: 10.1016/j.vaccine.2021.10.034 34702619

[31] Chen Y Wu Y Qin L Yu L Luo H Li Y . T-B cell epitope peptides induce protective immunity against Mycoplasma pneumoniae respiratory tract infection in BALB/c mice. Immunobiology. (2021) 226:152077. doi: 10.1016/j.imbio.2021.152077 33831654

[32] Mariuzza RA Wu D Pierce BG . Structural basis for T cell recognition of cancer neoantigens and implications for predicting neoepitope immunogenicity. Front Immunol. (2023) 14:1303304. doi: 10.3389/fimmu.2023.1303304 38045695 PMC10693334

[33] Xu L Xie N Liu Y Tang H Li T Peng J . Development of a novel multi-epitope vaccine against Streptococcus anginosus infection via reverse vaccinology approach. Immunology. (2025) 175:339–58. doi: 10.1111/imm.13936 40267989

[34] Tamiya S Yoshikawa E Ogura M Kuroda E Suzuki K Yoshioka Y . Neutrophil-mediated lung injury both via TLR2-dependent production of IL-1α and IL-12 p40, and TLR2-independent CARDS toxin after Mycoplasma pneumoniae infection in mice. Microbiol Spectr. (2021) 9:e0158821. doi: 10.1128/spectrum.01588-21 34937175 PMC8694186

[35] Ye T Jiao Z Li X He Z Li Y Yang F . Inhaled SARS-CoV-2 vaccine for single-dose dry powder aerosol immunization. Nature. (2023) 624:630–8. doi: 10.1038/s41586-023-06809-8 38093012

[36] Nazari M Emamzadeh R Masoudi-Khoram N Nazari M . Immobilization of albumin binding domain (ABD) on Sepharose 4B and magnetic particle for efficient single-step purification of human serum albumin. J Chromatogr B Analytical Technol Biomed Life Sci. (2025) 1261:124655. doi: 10.1016/j.jchromb.2025.124655 40403581

[37] Gao X Liu J Xu K Hu J Xiao C Wang D . Immunoaffinity chromatography for the preparation of equine tetanus immunoglobulin F(ab')(2) for enhanced safety and efficacy. J Chromatogr B Analytical Technol Biomed Life Sci. (2025) 1259:124591. doi: 10.1016/j.jchromb.2025.124591 40286483

[38] Long H He G He J Du TF Feng P Zhu C . The protective effect and immunomodulatory ability of orally administrated Lacticaseibacillus rhamnosus GG against Mycoplasma pneumoniae infection in BALB/c mice. PloS One. (2024) 19:e0312318. doi: 10.1371/journal.pone.0312318 39453930 PMC11508164

[39] Hausner M Schamberger A Naumann W Jacobs E Dumke R . Development of protective anti-Mycoplasma pneumoniae antibodies after immunization of Guinea pigs with the combination of a P1-P30 chimeric recombinant protein and chitosan. Microb Pathogen. (2013) 64:23–32. doi: 10.1016/j.micpath.2013.07.004 23948467

[40] Schurwanz N Jacobs E Dumke R . Strategy to create chimeric proteins derived from functional adhesin regions of Mycoplasma pneumoniae for vaccine development. Infect Immun. (2009) 77:5007–15. doi: 10.1128/iai.00268-09 19667041 PMC2772511

[41] Fonseca-Aten M Ríos AM Mejías A Chávez-Bueno S Katz K Gómez AM . Mycoplasma pneumoniae induces host-dependent pulmonary inflammation and airway obstruction in mice. Am J Respir Cell Mol Biol. (2005) 32:201–10. doi: 10.1165/rcmb.2004-0197oc 15626776

[42] Hardy RD Jafri HS Olsen K Wordemann M Hatfield J Rogers BB . Elevated cytokine and chemokine levels and prolonged pulmonary airflow resistance in a murine Mycoplasma pneumoniae pneumonia model: a microbiologic, histologic, immunologic, and respiratory plethysmographic profile. Infect Immun. (2001) 69:3869–76. doi: 10.1128/iai.69.6.3869-3876.2001 11349053 PMC98411

[43] Matute-Bello G Downey G Moore BB Groshong SD Matthay MA Slutsky AS . An official American Thoracic Society workshop report: features and measurements of experimental acute lung injury in animals. Am J Respir Cell Mol Biol. (2011) 44:725–38. doi: 10.1165/rcmb.2009-0210st 21531958 PMC7328339

[44] Cimolai N Taylor GP Mah D Morrison BJ . Definition and application of a histopathological scoring scheme for an animal model of acute Mycoplasma pneumoniae pulmonary infection. Microbiol Immunol. (2013) 36:465–78. doi: 10.1111/j.1348-0421.1992.tb02045.x 1513263

[45] Bielinska AU O'Konek JJ Janczak KW Baker JR Jr. Immunomodulation of TH2 biased immunity with mucosal administration of nanoemulsion adjuvant. Vaccine. (2016) 34:4017–24. doi: 10.1016/j.vaccine.2016.06.043 27317451 PMC4962973

[46] Ayivi-Tosuh SM Yang J Yang Y Li X Yang F Jiao L . Structure analysis of a non-esterified homogalacturonan isolated from Portulaca oleracea L. and its adjuvant effect in OVA-immunized mice. Int J Biol Macromol. (2021) 177:422–9. doi: 10.1016/j.ijbiomac.2021.02.142 33631260

[47] Wasan EK Syeda J Strom S Cawthray J Hancock RE Wasan KM . A lipidic delivery system of a triple vaccine adjuvant enhances mucosal immunity following nasal administration in mice. Vaccine. (2019) 37:1503–15. doi: 10.1016/j.vaccine.2019.01.058 30739796

[48] Meng Q Li P Li Y Chen J Wang L He L . Broncho-vaxom alleviates persistent allergic rhinitis in patients by improving Th1/Th2 cytokine balance of nasal mucosa. Rhinology. (2019) 57:451–9. doi: 10.4193/rhin19.161 31403136

[49] Victora GD Nussenzweig MC . Germinal centers. Annu Rev Immunol. (2022) 40:413–42. doi: 10.1146/annurev-immunol-120419-022408 35113731

[50] Kaplonek P Yao L Reppe K Voß F Kohler T Ebner F . A semisynthetic glycoconjugate provides expanded cross-serotype protection against Streptococcus pneumoniae. Vaccine. (2022) 40:1038–46. doi: 10.1016/j.vaccine.2021.12.068 35033388

[51] Hoffman C Eby J Gray M Heath Damron F Melvin J Cotter P . Bordetella adenylate cyclase toxin interacts with filamentous haemagglutinin to inhibit biofilm formation *in vitro*. Mol Microbiol. (2017) 103:214–28. doi: 10.1111/mmi.13551 27731909 PMC5218874

[52] Spuesens EBM Oduber M Hoogenboezem T Sluijter M Hartwig NG van Rossum AMC . Sequence variations in RepMP2/3 and RepMP4 elements reveal intragenomic homologous DNA recombination events in Mycoplasma pneumoniae. Microbiology. (2009) 155:2182–96. doi: 10.1099/mic.0.028506-0 19389769

[53] Kenri T Suzuki M Sekizuka T Ohya H Oda Y Yamazaki T . Periodic genotype shifts in clinically prevalent Mycoplasma pneumoniae strains in Japan. Front Cell Infect Microbiol. (2020) 10. doi: 10.3389/fcimb.2020.00385 32850484 PMC7424021

[54] Nilsson SS Fährmann L Sundelin T Nielsen L . Detection of Mycoplasma pneumoniae and Chlamydia pneumoniae using a laboratory-developed high-throughput test for the Panther Fusion system. Diagn Microbiol Infect Dis. (2025) 112:116786. doi: 10.1016/j.diagmicrobio.2025.116786 40054417

[55] Enriquez AS Avalos RD Parekh D Cooper CL Morrow G Geisbert TW . Mapping the antibody response to Lassa virus vaccination of non-human primates. EBioMedicine. (2025) 114:105673. doi: 10.1016/j.ebiom.2025.105673 40168843 PMC11999075

[56] Eshak F Goupil-Lamy A . Advancements in nanobody epitope prediction: A comparative study of AlphaFold2Multimer vs AlphaFold3. J Chem Inf Model. (2025) 65:1782–97. doi: 10.1021/acs.jcim.4c01877 39927847

[57] Besser H Yunger S Merhavi-Shoham E Cohen CJ Louzoun Y . Level of neo-epitope predecessor and mutation type determine T cell activation of MHC binding peptides. J Immunother Cancer. (2019) 7:135. doi: 10.1186/s40425-019-0595-z 31118084 PMC6532181

[58] Li G Fan L Wang Y Huang L Wang M Zhu C . High co-expression of TNF-α and CARDS toxin is a good predictor for refractory Mycoplasma pneumoniae pneumonia. Mol Med. (2019) 25:38. doi: 10.1186/s10020-019-0105-2 31399022 PMC6688229

[59] Sreenath K Li L Molotsky D Ayo J Baseman JB Kannan TR . Dual mode of delivery of Mycoplasma pneumoniae CARDS toxin determines the toxin trafficking pathway and cytotoxicity in host cells. mBio. (2026) 17:e0264025. doi: 10.1128/mbio.02640-25 41313181 PMC12802180

[60] Thomas R Qiao S Yang X . Th17/Treg imbalance: Implications in lung inflammatory diseases. Int J Mol Sci. (2023) 24:4865. doi: 10.3390/ijms24054865 36902294 PMC10003150

[61] Feng P Hu R Long H He J Xia Y Yang J . A multi-epitope vaccine targeting Mycoplasma pneumoniae P1 adhesin and CARDS toxin confers synergistic protection by adhesion inhibition and toxin neutralization. Sci Data Bank. (2026). doi: 10.57760/sciencedb.27991

