# Supplementary material for: A multi-epitope vaccine targeting Mycoplasma pneumoniae P1 adhesin and CARDS toxin confers synergistic protection by adhesion inhibition and toxin neutralization
Source: Front Immunol. 2026 Sep 22;17:1930950. doi: 10.3389/fimmu.2026.1930950 (PMC13638606; doi:10.3389/fimmu.2026.1930950)

# Flow cytometry gating strategy

## GCB and MBCs in the spleen 14 days post-final immunization.

## Flow Cytometric Gating Strategy for GCB and MBC:

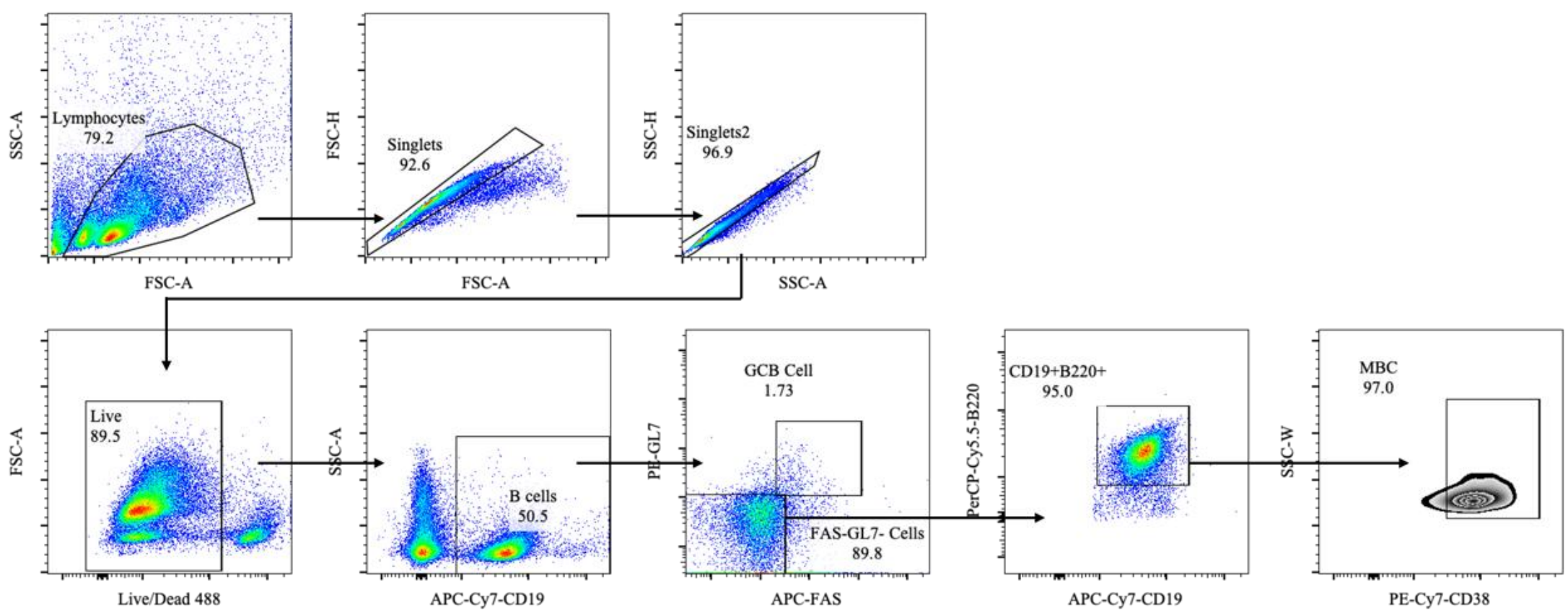

**Murine splenic T cells in 14 days after the last vaccine immunization.**

**Flow Cytometric Gating Strategy for TN, Teff and TCM:**

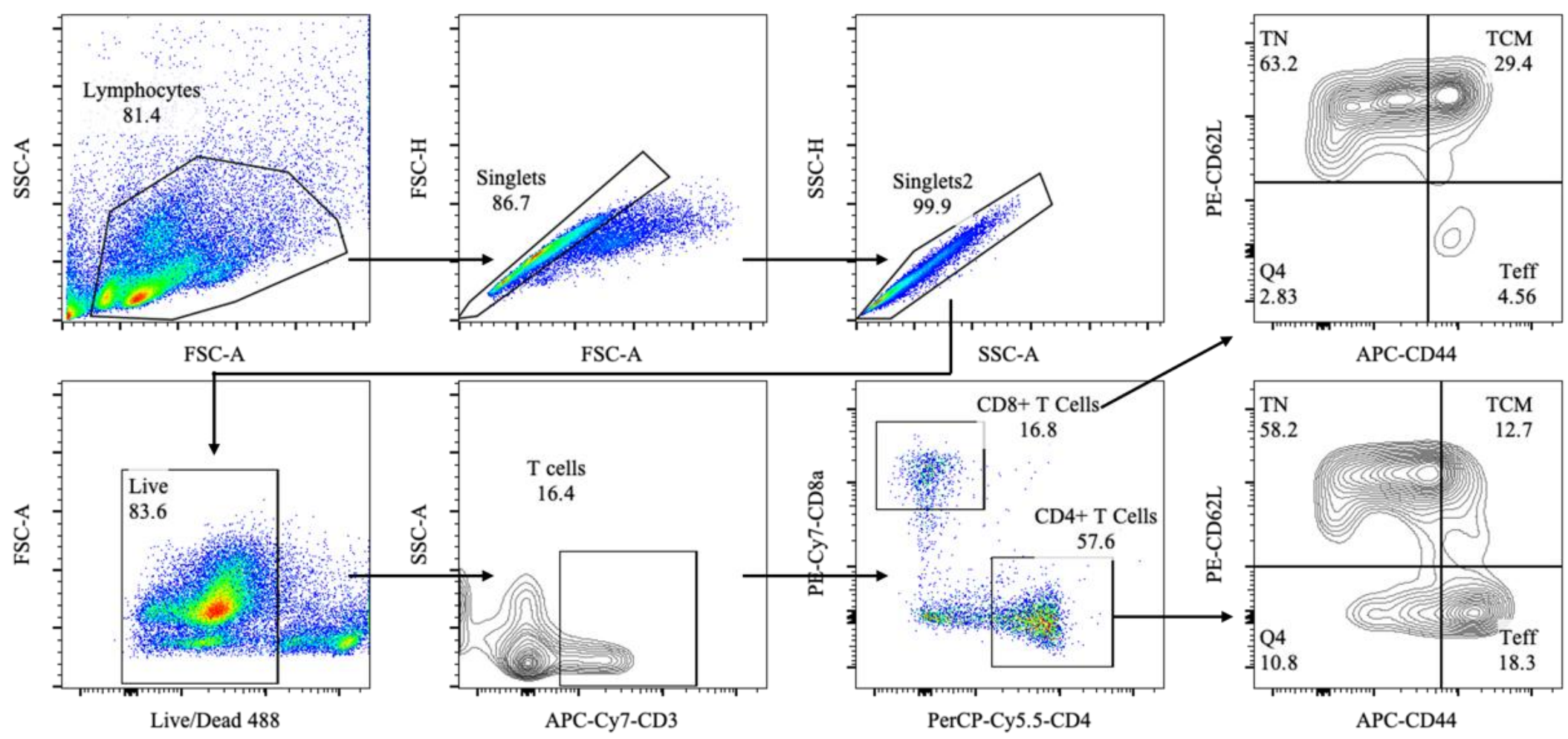

**T cells in mouse spleen 14 days post-vaccination.**

**Flow Cytometric Gating Strategy for Th1, Th2 and CTL.**

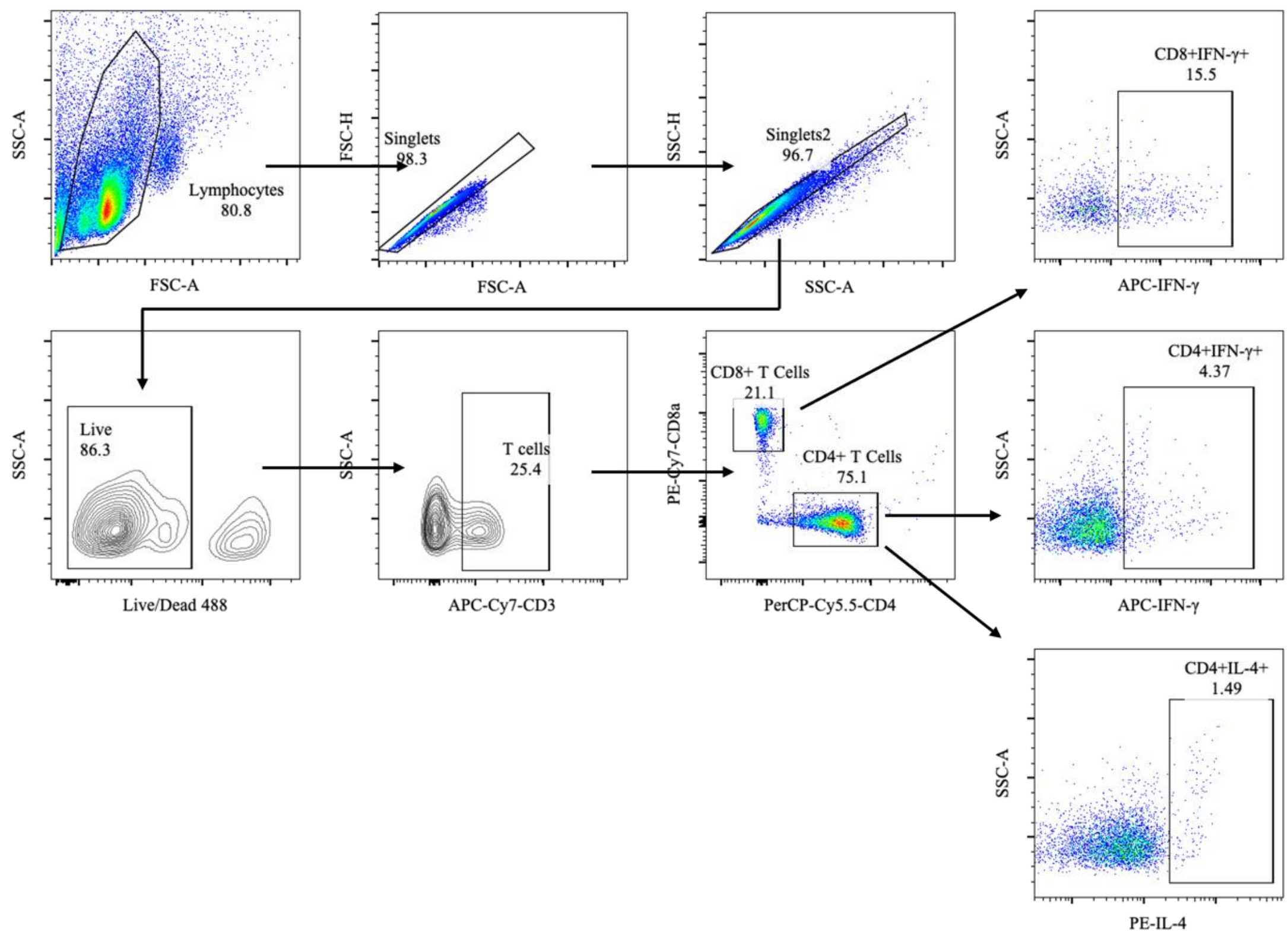

**Intracellular cytokines in pulmonary T cells of mice at 4dpi.**

**Flow Cytometric Gating Strategy for Th1, Th2 and CTL cells:**

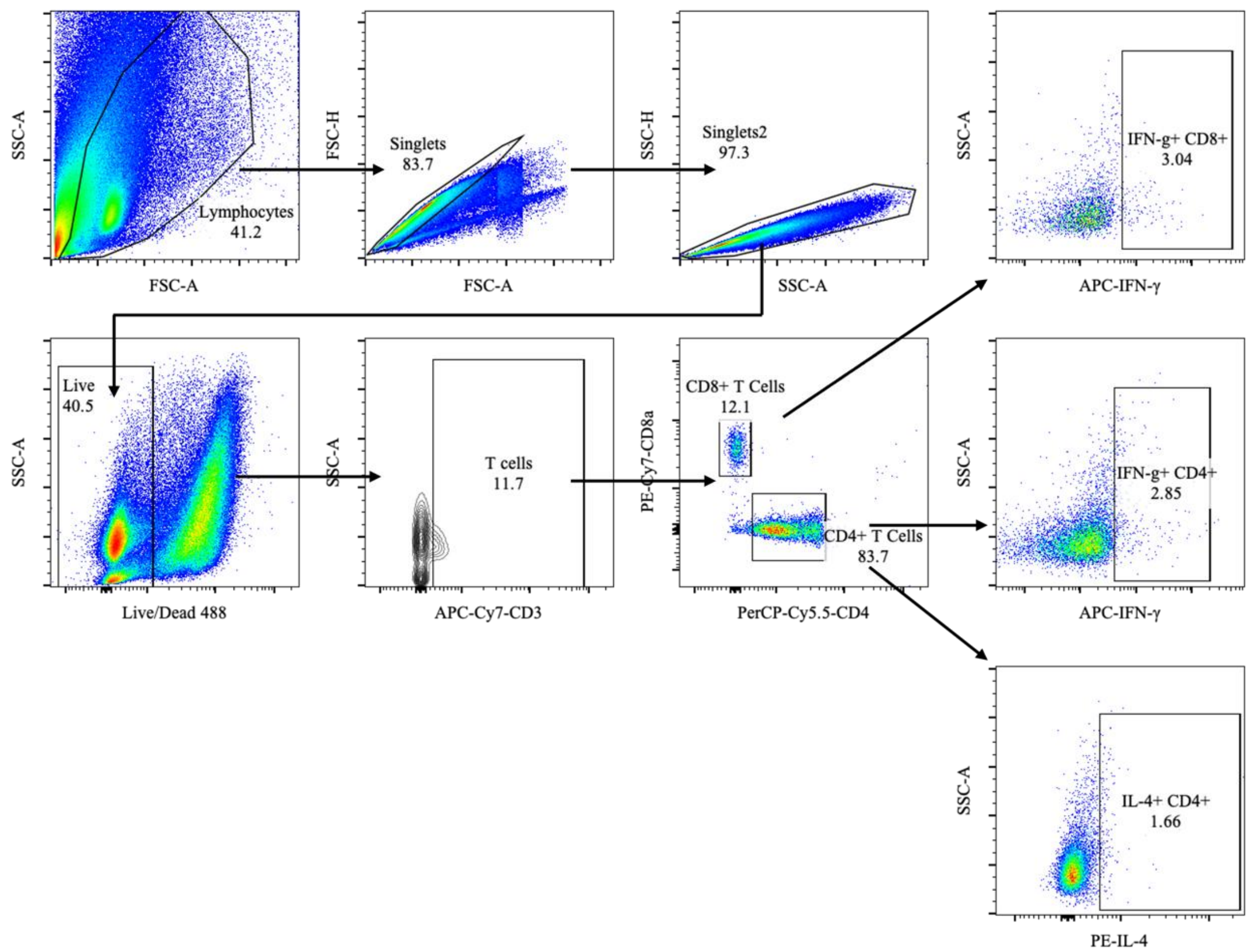

Supplement: Supplementary file 1 [file DataSheet1.pdf]
