# Supplementary material for: A multi-epitope vaccine targeting Mycoplasma pneumoniae P1 adhesin and CARDS toxin confers synergistic protection by adhesion inhibition and toxin neutralization
Source: Front Immunol. 2026 Sep 22;17:1930950. doi: 10.3389/fimmu.2026.1930950 (PMC13638606; doi:10.3389/fimmu.2026.1930950)

Supplementary Tables

Table. S1 Th epitope prediction of P1C and CARDS proteins

Protein	Alleles	Start	End	Sequence (peptide)	Score
P1C	HLA-DRB4*01:01	1137	1151	GLQLTGLDASDATQR	0.6336
P1C	HLA-DRB3*02:02	1219	1234	ASAYKPNTSSGQTQS	0.6406
P1C	HLA-DRB1*07:01	1238	1252	SPYLHLVKPKKVTQS	0.7352
P1C	HLA-DRB1*07:01	1415	1429	QIDFNRLFTHPVTDL	0.4697
P1C	HLA-DRB1*03:01	1440	1455	YIPLFIDIPASVNPKM	0.6514
CARDS	HLA-DRB1*07:01	2	16	PNPVRFVYAVDLRSP	0.8408
CARDS	HLA-DRB1*15:01	322	336	RICLKDLTGAQISLS	0.7903
CARDS	HLA-DRB1*15:01	337	351	LTAFTTQYAGQLKVH	0.7441
CARDS	HLA-DRB1*03:01	471	485	GQIFYDLKTSKIFFV	0.9372

Table. S2 B cell epitope prediction of P1C and CARDS proteins

Protein	Start	End	Sequence (peptide)	Length
P1C	1056	1073	KYFGNTRAGGSGSNTTS	17
P1C	1066	1082	SGSNTTSSPGIGFKIP	16
P1C	1151	1166	RALIWAPRPWAAFRGS	16
P1C	1312	1328	GGGSSGSGQSGVDLSP	16
P1C	1398	1413	GQTADTGPQSVKFKSP	16
CARDS	78	93	LYEIRADQHFYNARAT	16
CARDS	454	469	YYTPQLSGWSYQMKTP	16
CARDS	562	577	SRWGGWYSTYDKVESN	16

Table. S3 Screening of dominant antigenic epitopes of P1C and CARDS proteins

Protein	Sequence (peptide)	Immunogenicity Score	Antigenicity Score	Allergenicity	Toxin
P1C	GLQLTGLDASDATQR	-0.06665	1.0272	ALLERGEN	Non-Toxin
P1C	SPYLHLVKPKKVTQS	-0.58832	0.3727	ALLERGEN	Non-Toxin
P1C	QIDFNRLFTHPVTDL	0.41538	-0.1727	ALLERGEN	Non-Toxin
P1C	ASAYKPNTSSGQTQS	-0.61348	1.5249	NON-	Non-Toxinn
P1C	YIPLFIDIPASVNPKM	0.27316	1.5681	ALLERGEN	Non-Toxin
CARDS	PNPVRFVYAVDLRSP	0.27542	1.0074	NON-	Non-Toxin
CARDS	RICLKDLTGAQISLS	-0.25258	1.1559	NON-	Non-Toxin
CARDS	LTAFTTQYAGQLKVH	-0.12032	0.2199	ALLERGEN	Non-Toxin
CARDS	GQIFYDLKTSKIFFV	-0.08262	-0.0805	ALLERGEN	Non-Toxin

Table. S4 Composition of antigenic epitopes in P1Tx proteins

Protein	Start	End	Sequence (peptide)	Type
P1C	1137	1151	GLQLTGLDASDATQR	HTL
P1C	1238	1252	SPYLHLVKPKKVTQS	HTL
P1C	1415	1429	QIDFNRLFTHPVTDL	HTL
CARDS	2	16	PNPVRFVYAVDLRSP	HTL
CARDS	322	336	RICLKDLTGAQISLS	HTL
CARDS	337	351	LTAFTTQYAGQLKVH	HTL
CARDS	471	485	GQIFYDLKTSKIFFV	HTL
P1C	1151	1166	RALIWAPRPWAAFRGS	B Cell
P1C	1313	1328	GGGSSGSGQSGVDLSP	B Cell
P1C	1398	1413	GQTADTGPQSVKFKSP	B Cell
CARDS	78	93	LYEIRADQHFYNARAT	B Cell
CARDS	454	469	YYTPQLSGWSYQMKTP	B Cell
CARDS	562	577	SRWGGWYSTYDKVESN	B Cell

Table. S5 The linear B-cell epitopes prediction of P1Tx proteins

Start	End	Peptide	Number of residues	Score
164	209	PRPWAAFRGSKKGGGSSGSGQSGVDLSPKKG QTADTGPQSVKFKSP	46	0.843
1	53	GLQLTGLDASDATQRGPGPGSPYLHLVKPKK VTQSDKLDDDLKNLLDGPGPGQ	53	0.72
66	74	DLGPGPGPN	9	0.613
87	93	PEGPGPG	7	0.564
225	248	RATKKYYTPQLSGWSYQMKTPKKS	24	0.542

Table. S6 The conformational B-cell epitopes prediction of P1Tx proteins

No.	Residues	Number of residues	Score
1	193-204	12	0.908
2	171-192	22	0.901
3	001-005	15	0.886
4	167-170	4	0.818
5	16-22	7	0.734
6	31-54	24	0.655
7	226-229	4	0.623
8	206-209	4	0.604
9	65-74	10	0.592
	230-239		
	241-248		
10	251-253	29	0.545
	255-256		
	258-263		
11	23-30	8	0.541
	87		
12	89-93	7	0.538
	109		

Table. S7 Antigenicity, allergenicity, toxicity and physicochemical properties of P1-Tx protein

Protein	P1Tx
ToxinPred	Non-Toxin
VaxiJenscore	0.7406
ANTIGENpro	0.932809
AllerTOP v2.0	Non-allergen
VirulentPred	Virulent
Molecular weight	28511.24
pI	9.81
Half-life (in vitro)	30 hours
Half-life (in vivo)	>10 hours
Instability index	30.86
Aliphatic index	61.56
Grand average of hydropathicity	-0.701

Supplementary Figures

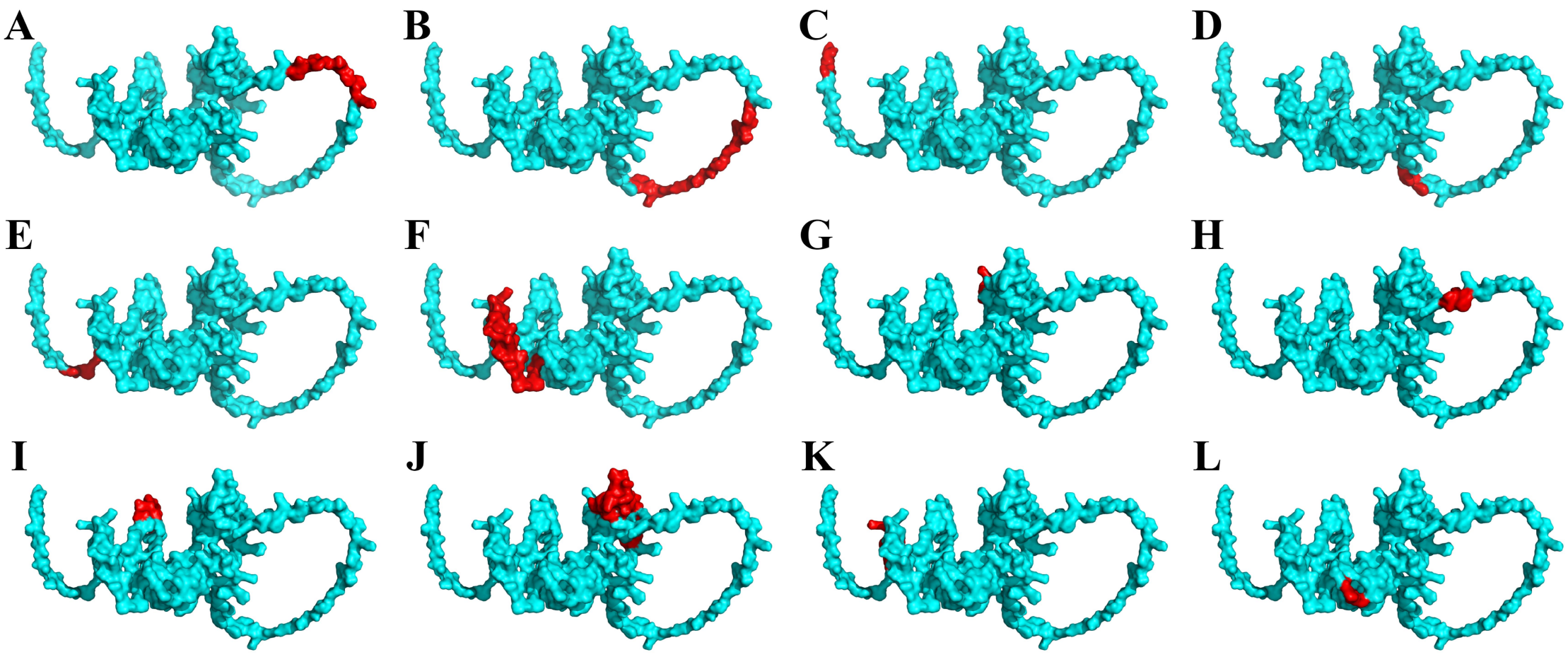

Figure.S1 The conformational B-lymphocyte epitopes present in the vaccine. The light red spheres showing epitopes containing: a) 12 residues (AA193-204) with 0.908; b) 22 residues (AA171-192) with 0.901; c) 15 residues (AA1-15) with 0.886; d) 4 residues (AA167-170) with 0.818; e) 7 residues (AA16-22) with 0.734; f) 24 residues (AA31-54) with 0.655; g) 4 residues (AA226-229) with 0.623; h) 4 residues (AA206-209) with 0.604; i) 10 residues (AA65-74) with 0.592; j) 29 residues (AA230-239, AA241-248, AA251-253, AA255-256, AA258-263) with 0.545; k) 8 residues (AA23-30) with 0.604; l) 7 residues (AA87, AA89-93, AA109) with 0.592.

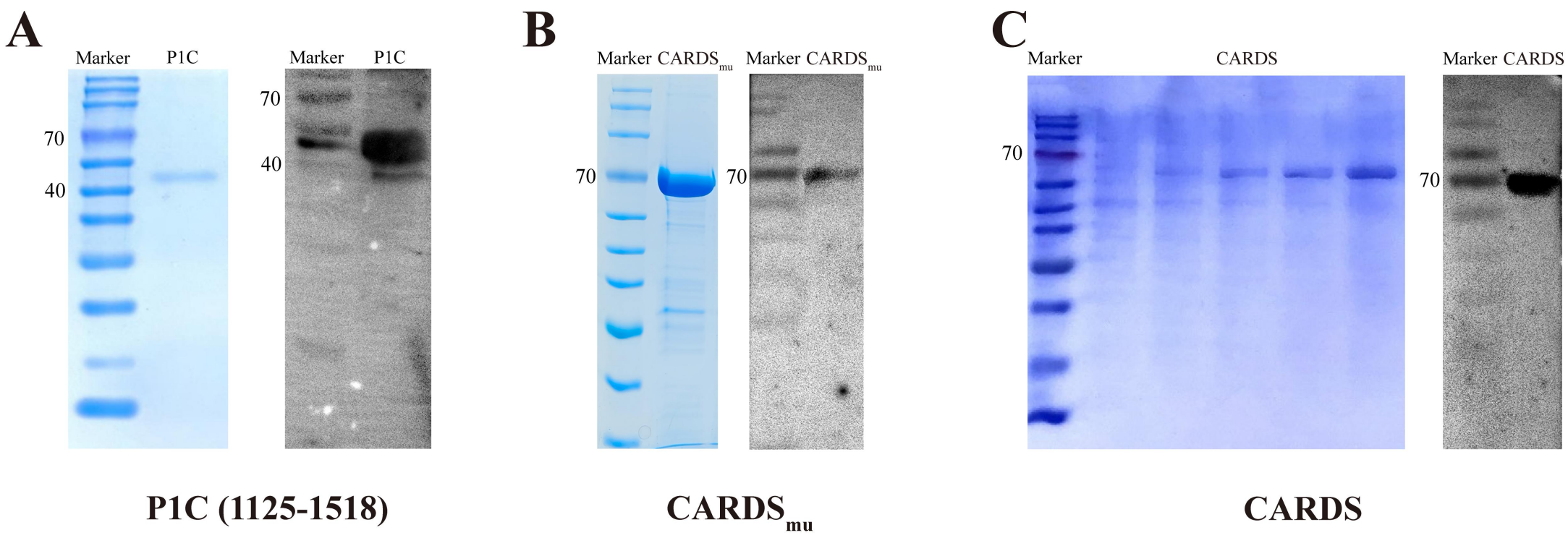

Figure.S2 Expression optimization and purification of P1C, CARDSmu and CARDS proteins.

A) P1C; B) CARDSmu; C) CARDS.

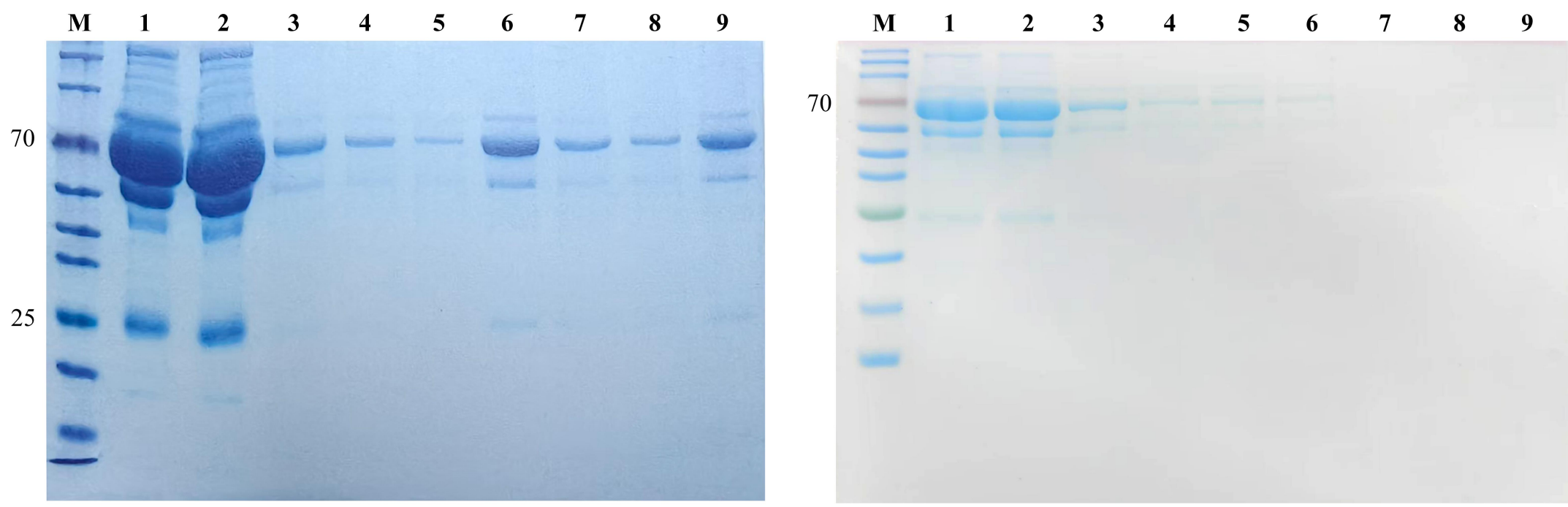

Figure.S3 SDS-PAGE analysis of specific antibody purification for P1Tx.

(Left) M: Marker; Lanes1, 2: serum (before and after purification); Lanes 3~5, 6~8: P1C and CARDS protein-specific serum antibody purification (three times). (Right) Lane 1, 2: serum (before and after purification); Lanes 3~9: P1Tx protein-specific serum antibody purification.

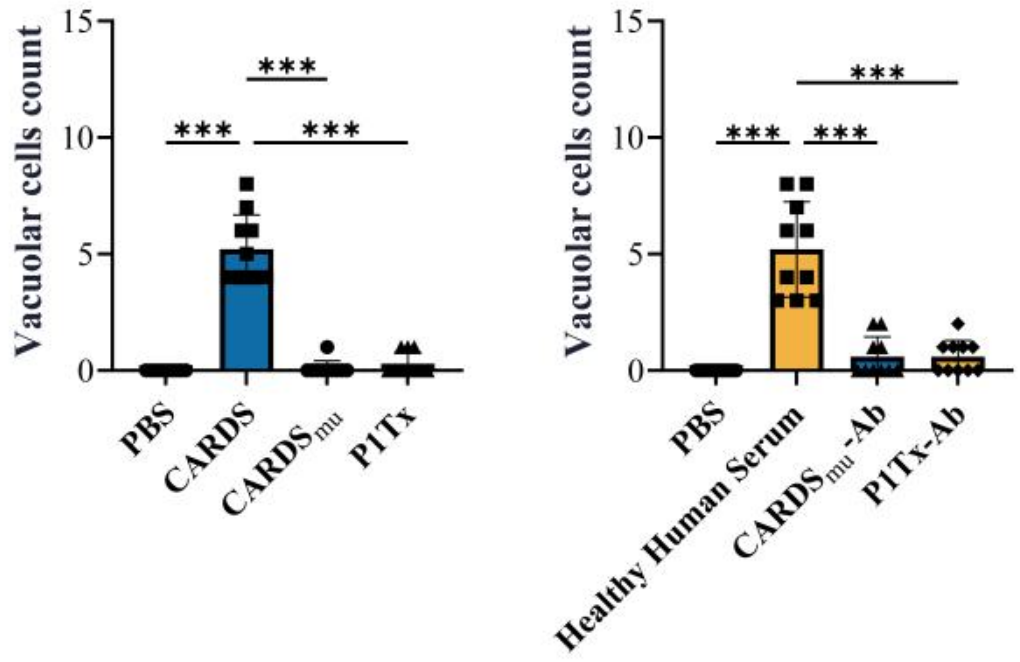

Figure.S4 Statistical analysis of vacuolating toxicity and neutralization.(Left) Vacuolating Toxicity; (Right) Neutralization of Vacuolating Toxicity. Statistical analysis was performed using the One-way ANOVA test (***$P < 0.001$, ** $P < 0.01$, * $P < 0.05$). Error bar, mean±S.D.

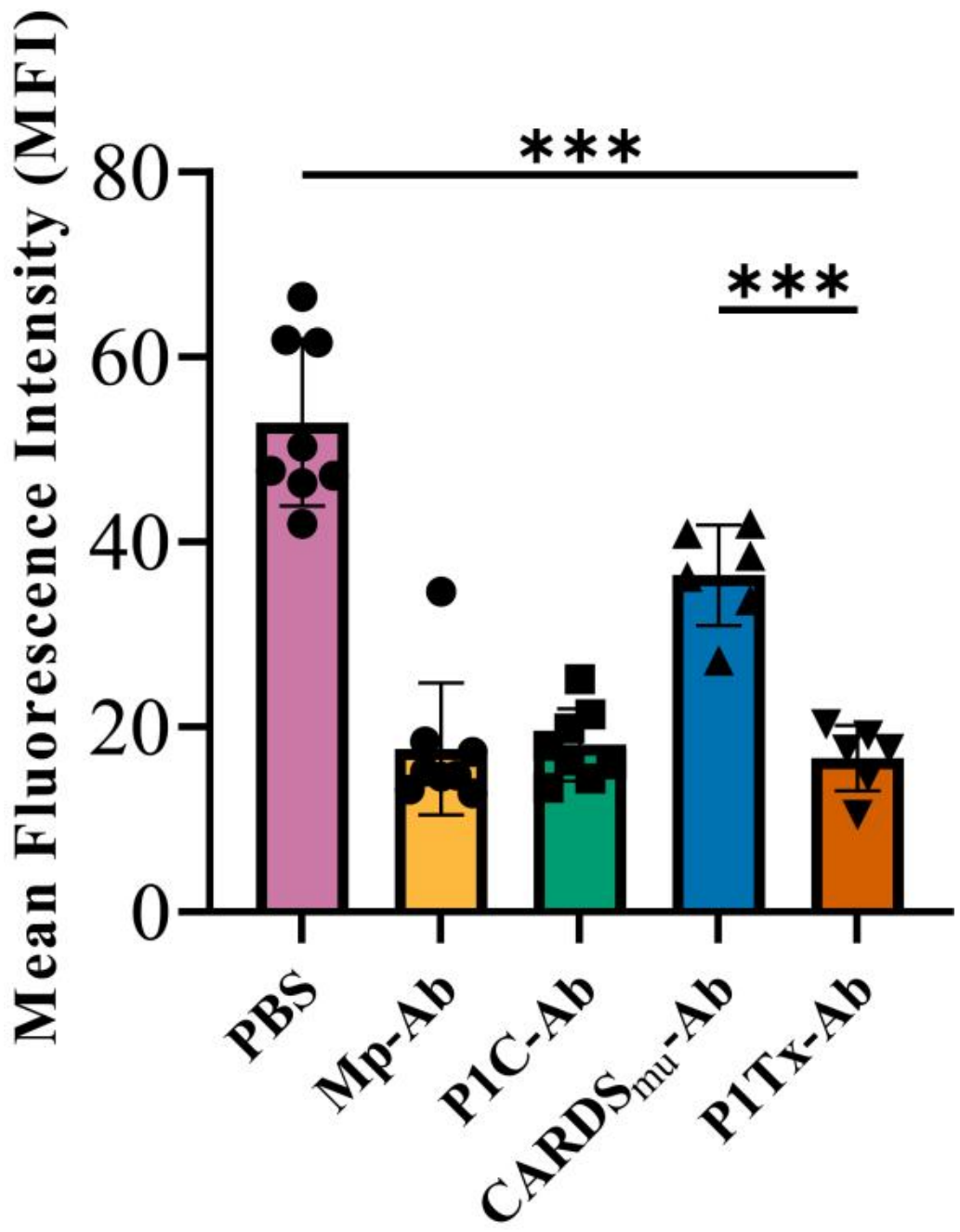

Figure. S5 MFI of IFA-based adhesion inhibition assay using immune serum.

Statistical analysis was performed using the One-way ANOVA test (****P* < 0.001, ** *P* < 0.01, * *P* < 0.05). Error bar, mean±S.D.

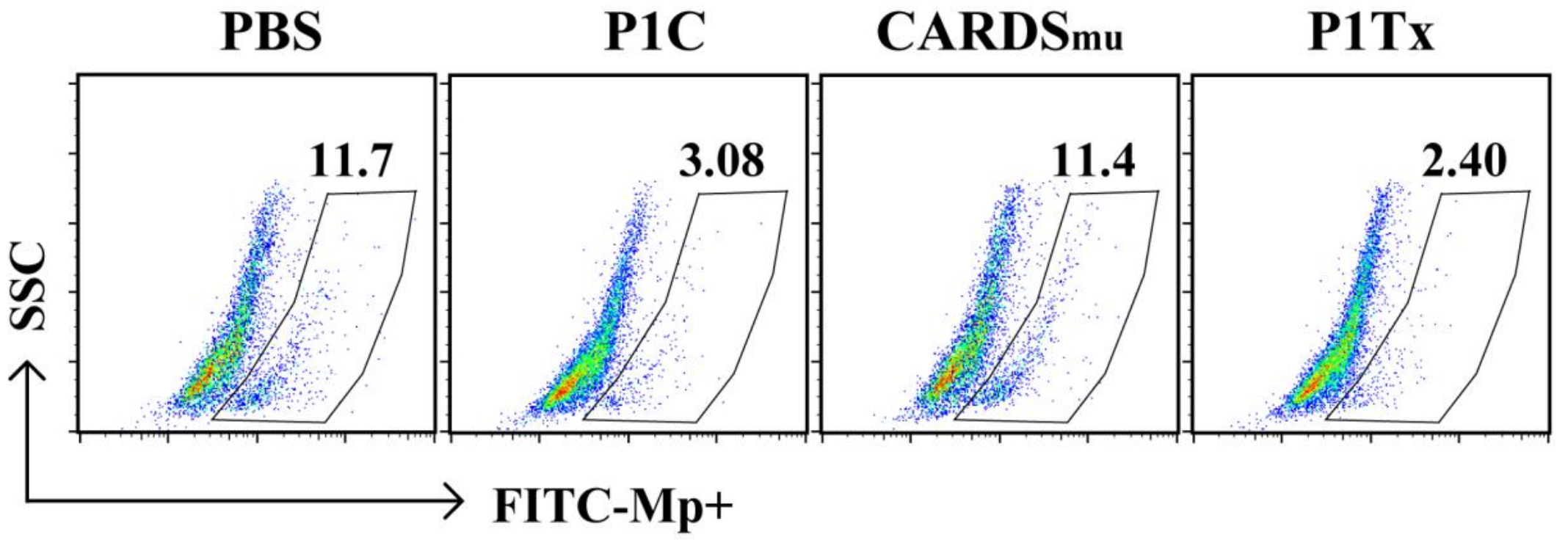

Figure.S6 Flow cytometry analysis of adhesion inhibition.

Representative flow data of adhesion inhibition mediated by mouse serum at 14 days post-final immunization.

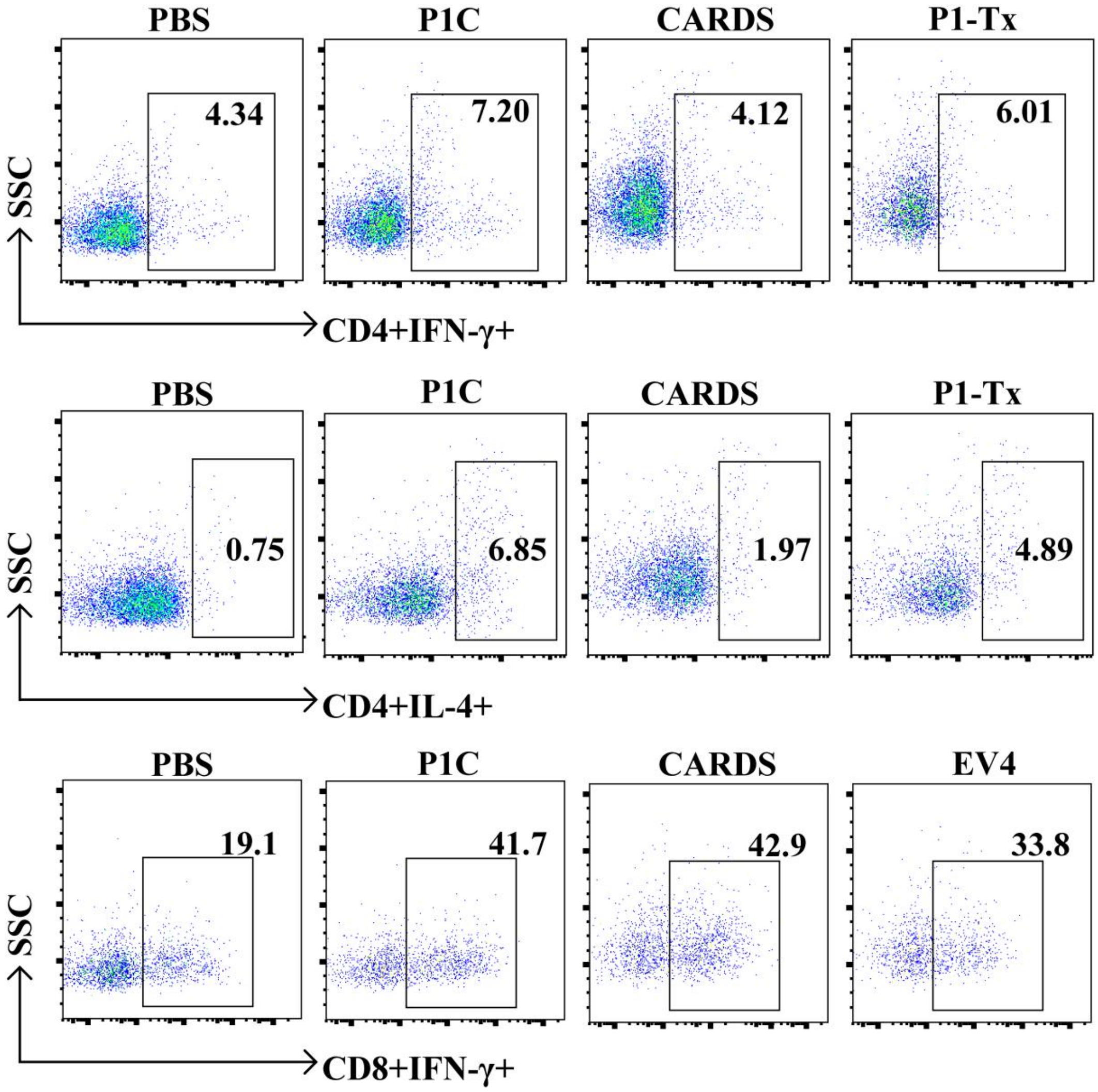

Figure.S7 Flow cytometry analysis of Intracellular cytokines of splenic lymphocytes at 14 days post-final immunization.

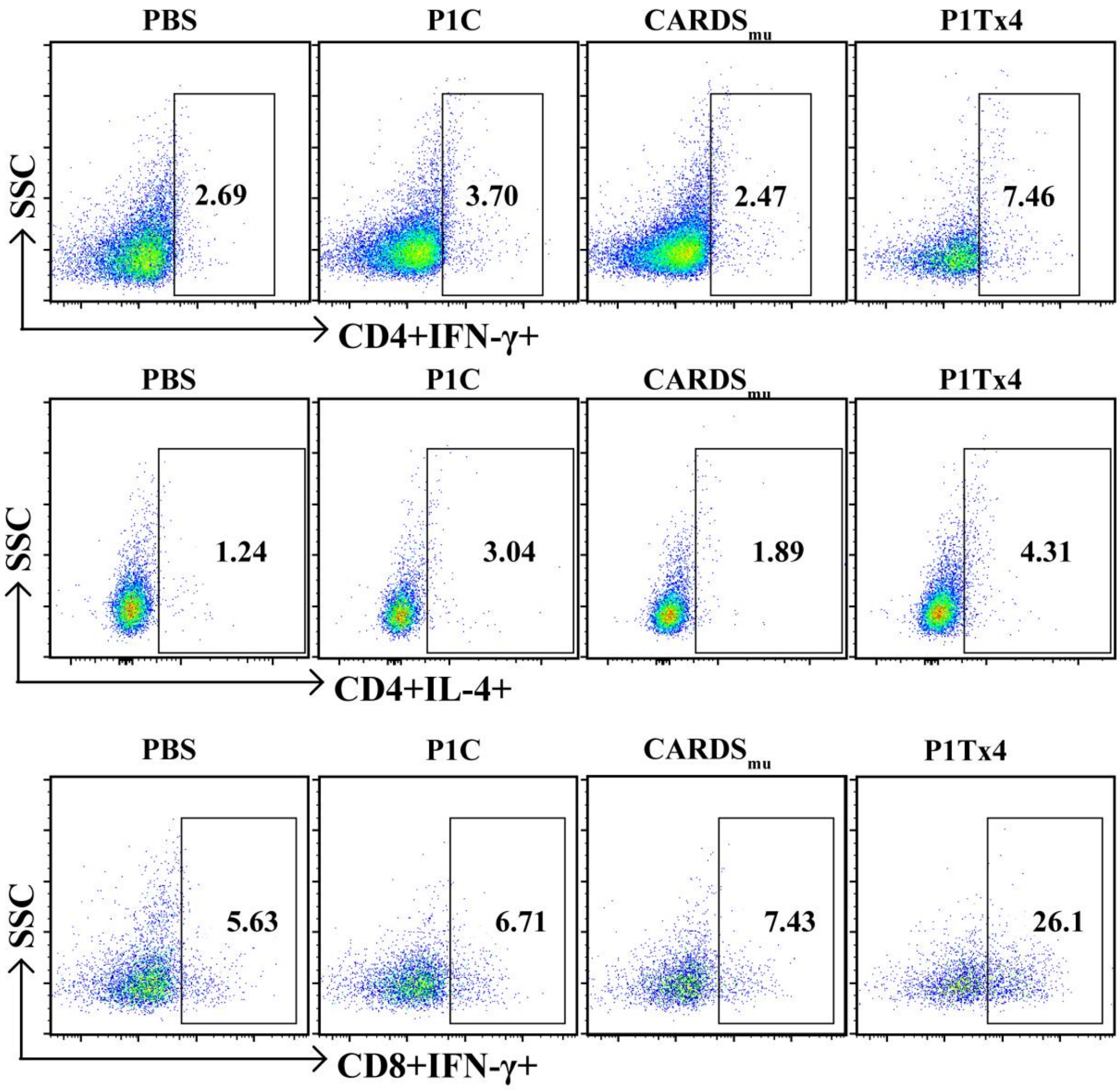

Figure.S8 Flow cytometry analysis of Intracellular cytokines of pulmonary lymphocytes on day 4 post-infection.

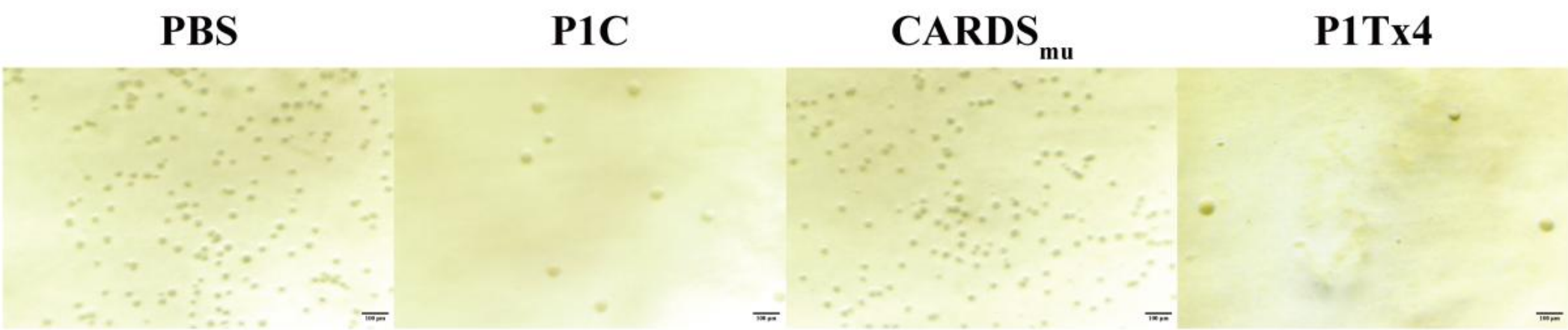

Figure.S9 Mp colonies cultured from the supernatant mouse pulmonary homogenate on day 4 post-infection. view of ×100 magnification, Scale bar: 50μm.

Flow cytometry gating strategy

GCB and MBCs in the spleen 14 days post-final immunization.

Flow Cytometric Gating Strategy for GCB and MBC:

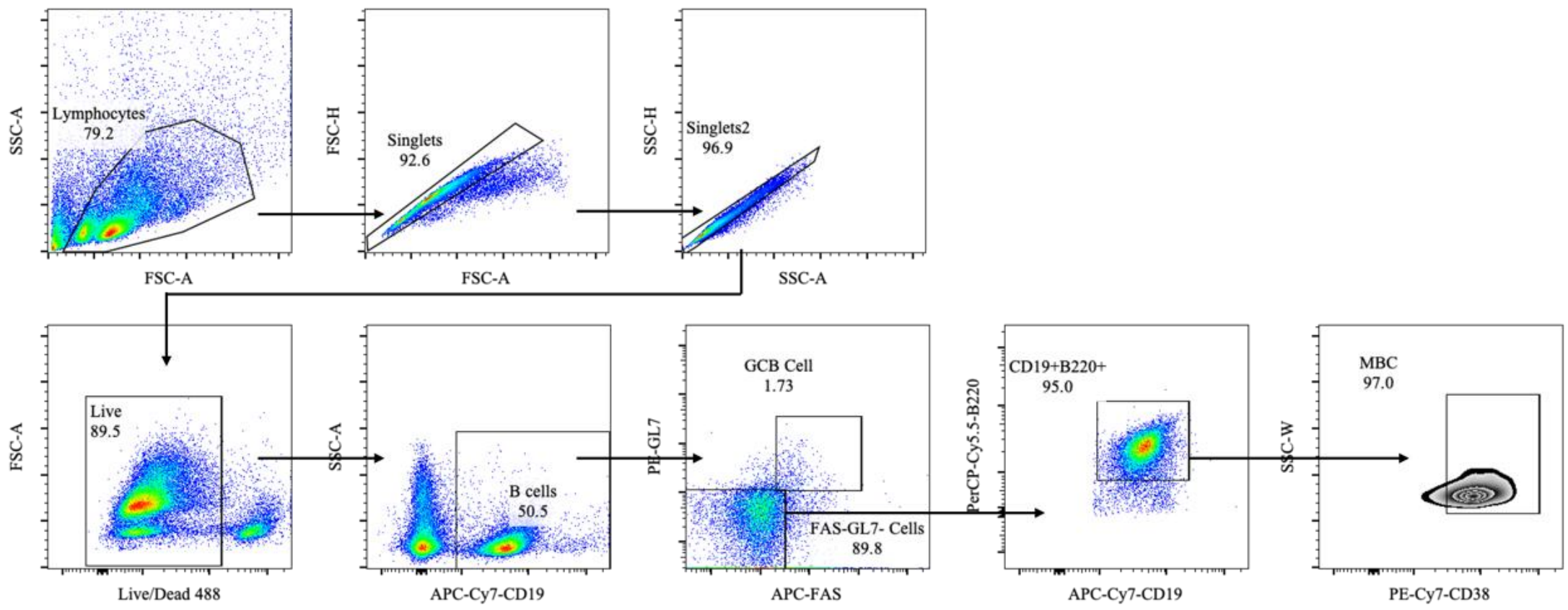

Murine splenic T cells in 14 days after the last vaccine immunization.

Flow Cytometric Gating Strategy for TN, Teff and TCM:

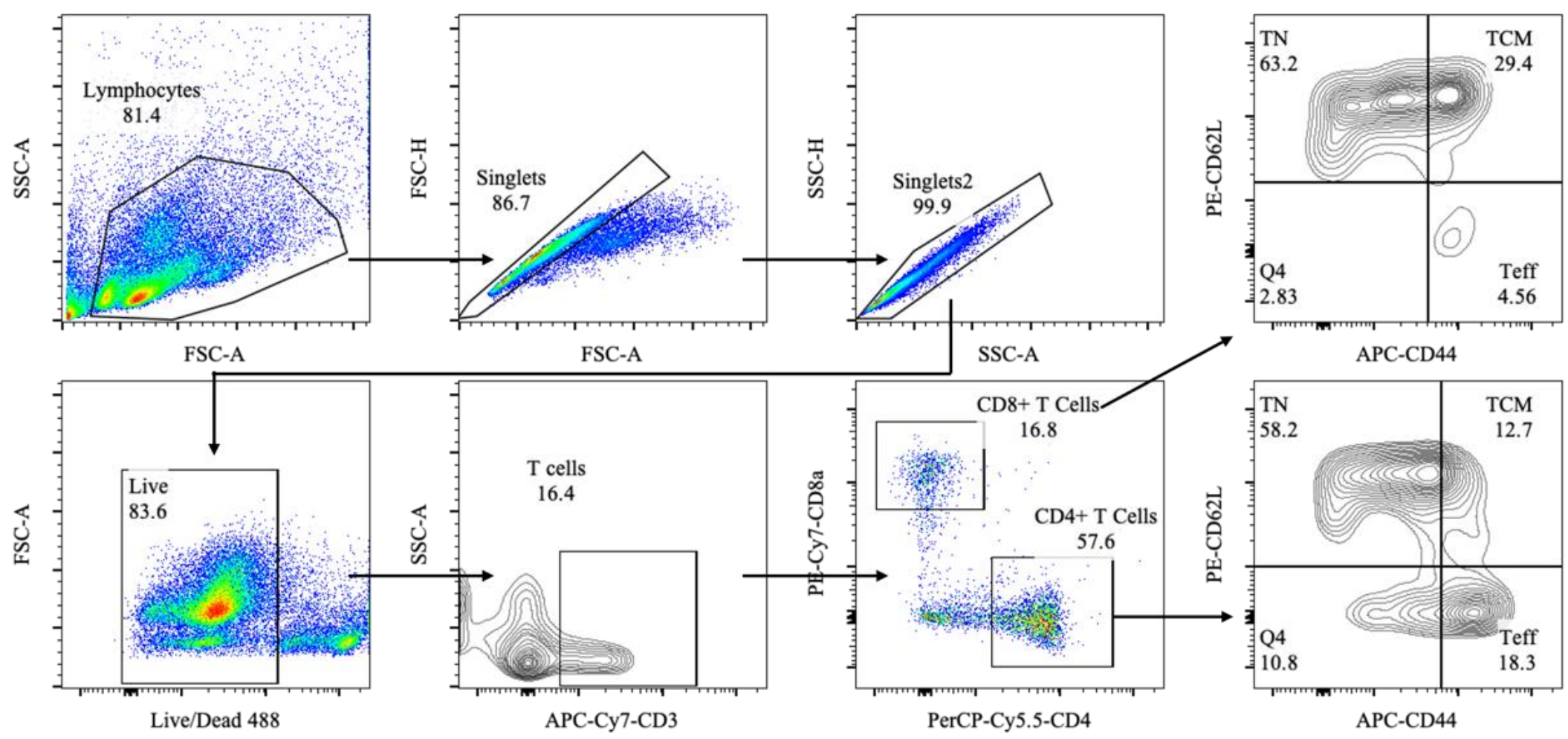

T cells in mouse spleen 14 days post-vaccination.

Flow Cytometric Gating Strategy for Th1, Th2 and CTL.

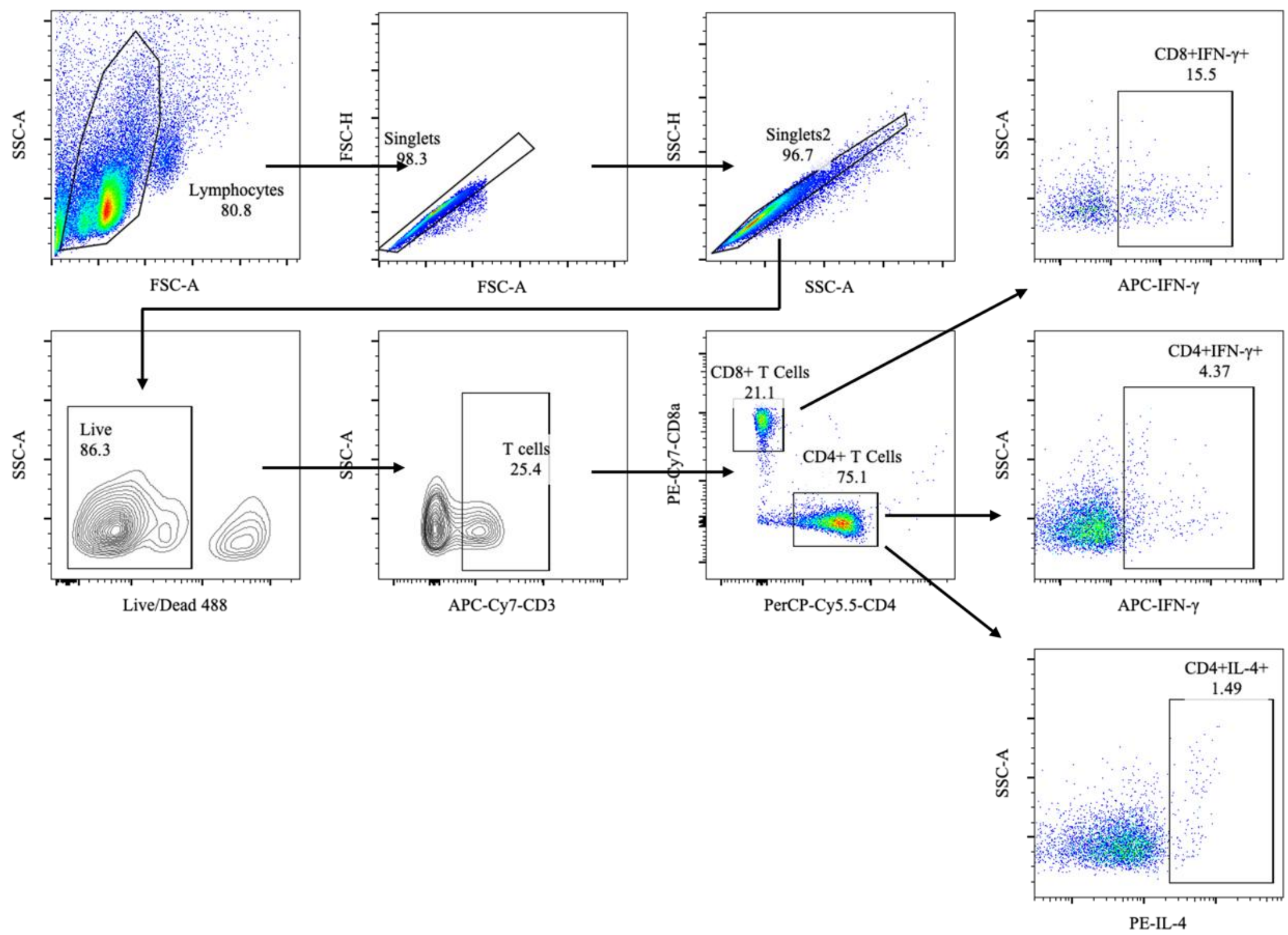

Intracellular cytokines in pulmonary T cells of mice at 4dpi.

Flow Cytometric Gating Strategy for Th1, Th2 and CTL cells:

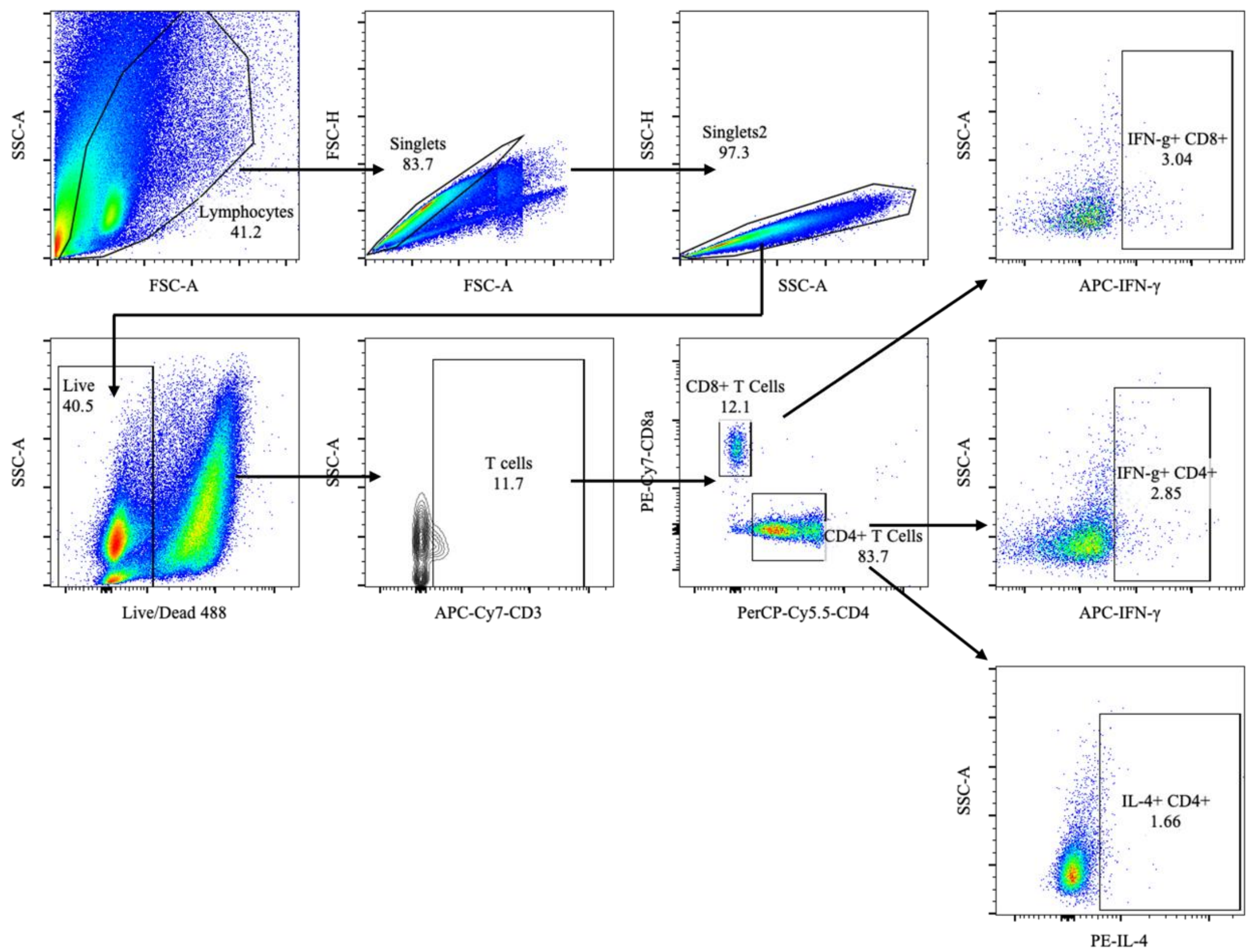

Supplement: Supplementary file 2 [file DataSheet2.pdf]
